# A heteroditopic macrocycle as organocatalytic nanoreactor for pyrroloacridinone synthesis in water

**DOI:** 10.3762/bjoc.15.152

**Published:** 2019-07-08

**Authors:** Piyali Sarkar, Sayan Sarkar, Pradyut Ghosh

**Affiliations:** 1 School of Chemical Sciences, Indian Association for the Cultivation of Science, 2A & 2B Raja S.C Mullick Road, Kolkata-700032, India

**Keywords:** heteroditopic macrocycle, organocatalyst, nanoreactor, pyrroloacridinones, sustainable chemistry, water

## Abstract

A heteroditopic macrocycle is reported as an efficient organocatalytic nanoreactor for the synthesis of diversely functionalized pyrroloacridinones in aqueous medium. A library of compounds was synthesized in a one-step pathway utilizing 10 mol % of the nanoreactor following a sustainable methodology in water with high yields.

## Introduction

Acridines are a renowned heterocyclic entity not only for their biological activities but also for their fluorescence and chemiluminescence properties. Acridine dyes (e.g., acridine orange, acridine yellow, etc.), are predominantly used in staining of apoptotic cells, the investigation of the morphology of nuclei, the direct counting of cultivable bacteria, DNA intercalators, etc. [1–4]. Pyrrole, another biologically active heterocyclic compound, when fused with acridines affords promising bioactive pyrroloacridinone moieties demonstrating antitumor, antifungal, anthelmintic, anticancer activities, etc. [5–7]. Although a few syntheses of these molecules have been reported in the literature, these generally consist of multistep protocols and require harsh acidic catalysts [5,8–12]. Moreover, only in a very few cases, the environmentally benign solvent water has been used [11].

Organocatalytic nanoreactors have emerged as an exciting area for novel organic syntheses, offering environmentally friendlier processes [13–18]. The distinct nanospace around the substrates, use of green solvents and catalyst recycling, makes them highly valuable in view of sustainable chemical applications. Thus, the development of organocatalytic nanoreactors with new features is indeed important to address greener organic syntheses.

On the other hand, the rational design of heterotopic macrocycles has attracted intense interest as efficient organocatalysts due to their variable interacting sites. In this context, substituted calixarenes, cyclodextrins, etc., substances known for their ability to encapsulate organic guest molecules, have been explored as organocatalysts over the last two decades [19–24]. Another class of macrocycles with multiple functional groups has been employed as alternative templates to induce the organic environment for catalysis via multiple weak interactions viz. π–π stacking, hydrogen bonding, etc. [25–29]. At the same time, several efforts have been made to develop environmentally oriented efficient organocatalysts with prominence “on-water conditions” [30–31]. It is known that an important condition for an organic reaction in water is the aggregation of the reactants and the catalyst by hydrophobic forces. Further, the potential of hydrogen-bond donation may be a vital issue for the stabilization of the activated complex by a hydrophobic catalyst [31]. Again, the efficiency of an organocatalyst could be enhanced by downsizing the catalyst at nanorange distribution (i.e., nanoreactor) [13–14]. This is because a nanoreactor allows for precise interactions with the reactants. In fact, encapsulation can result in the stabilization of a specific transition state by stripping it out from solvent molecules [13–14]. Hence, in aqueous medium, the syntheses of nanoreactors with a covalent organic framework is important, which is indeed a difficult task due to their large aggregation tendency. Herein we have extensively explored a multifunctional macrocycle (BATA-MC), comprising bis-amide and tris-amine functionalities as H-bond donor/acceptor moieties, and parallel benzene moieties for aromatic π–π stacking interactions as an organocatalytic nanoreactor for organic transformations, in particular, for the synthesis of biologically important highly substituted pyrroloacridines in the ecofriendly solvent water.

## Results and Discussion

### Synthesis of the macrocycle

**Figure 1:**
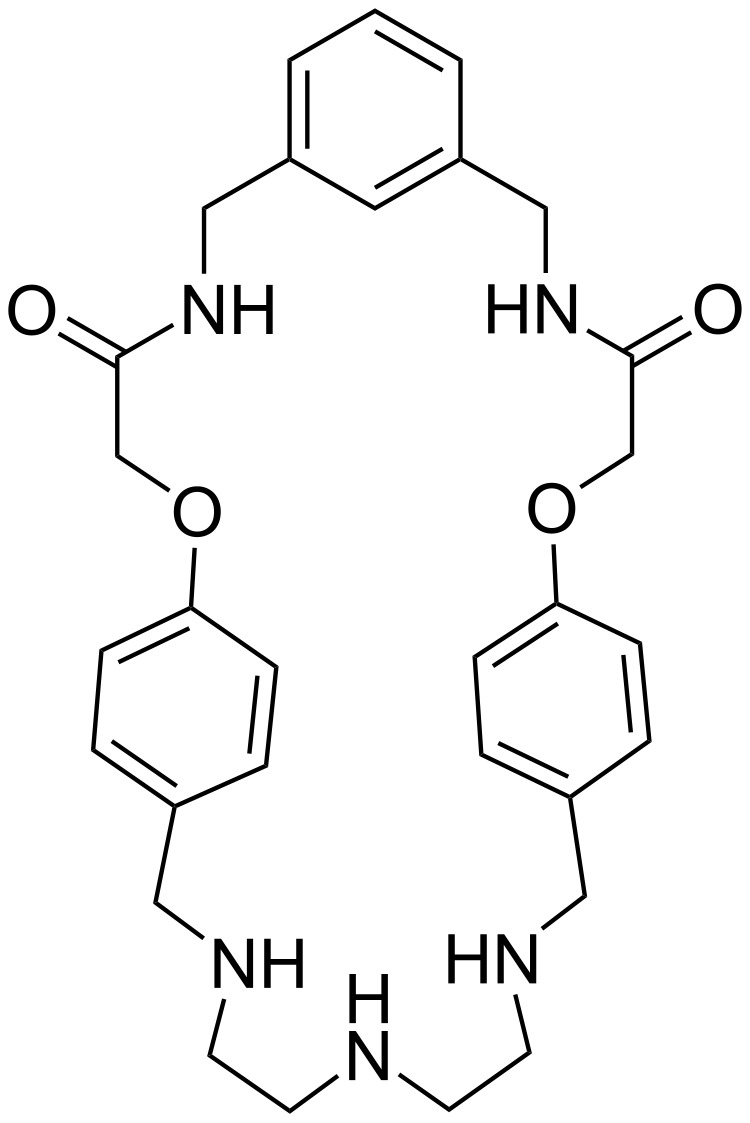
Bis-amido-tris-amine macrocycle BATA-MC.

Considerable efforts in synthesizing multifunctional macrocycles have been dedicated lately for the construction of supramolecular assemblies like molecular rotors (pseudorotaxane, rotaxane, catenane), molecular switches, molecular shuttles, etc. [32–43]. Furthermore, macrocycles have been applied in the area of ion–ion pair recognition and heterometallic complex formation [44–50]. The multifunctional macrocycle BATA-MC (Figure 1) has been successfully applied as a wheel in the molecular rotors fields [51]. Considering its multiple binding sites (π-interactions, H-bonding site, cavity), we investigated the catalytic activity of this macrocycle. BATA-MC was synthesized following our previously reported procedure [51]. The synthesis involves three steps utilizing 1,3-diaminomethylbenzene, chloroacetyl chloride, 4-hydroxybenzaldehyde and diethylenetriamine as easily available starting materials. The desired product BATA-MC was isolated as white solid with 80–81% yield in the final step (Supporting Information File 1, Scheme S1).

### Preparation and properties of the nanoreactor

**Figure 2:**
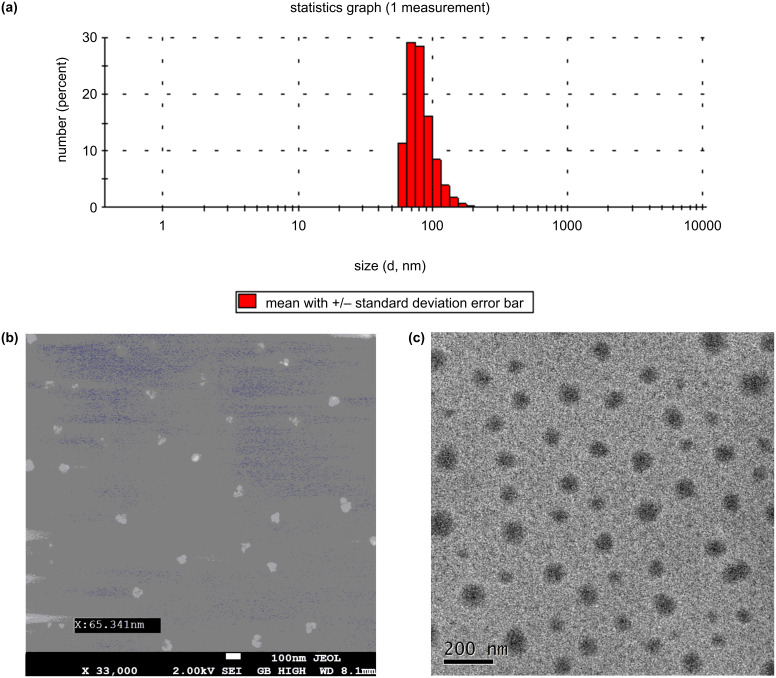
(a) Number distribution plot with particle size in DLS, (b) SEM image and (c) TEM image showing the nanoranged particle distribution of the macrocycle.

To prepare nanoranged particles of BATA-MC the synthesized macrocycle was suspended in water followed by ultrasonication for five minutes. Then, the dispersion of BATA-MC in water was subjected to various studies like SEM, TEM and DLS experiments. The DLS data revealed that the dispersed particles size in the aqueous phase varied from ≈60 nm to ≈105 nm (Figure 2a). Interestingly, when the same dispersion was used for the analysis of the particle size distribution on a solid surface, i.e., in SEM and TEM, the diameters were also found to be in the range of 70–100 nm (Figure 2b and 2c). So, these studies supported the nanoranged dispersion of the macrocycle, which indeed follows the basic characteristic of a nanoreactor.

### Optimization of the reaction conditions for the synthesis of pyrroloacridinones

The organocatalytic activity of the BATA-MC nanoreactor was then explored in the synthesis of pyrroloacridines. To optimize the reaction conditions, we chose the synthesis of **4a** as the model reaction. Thus, we performed the reaction of isatin (**1a**, 1 mmol), 5,5-dimethylcyclohexane-1,3-dione (**2**, 1 mmol) and *p*-toluidine (**3a**, 1 mmol) in water under different conditions (Table 1). Importantly, the catalyst BATA-MC showed excellent efficacy affording the desired product **4a** in high yields in aqueous medium (Table 1, entries 2–7). To examine the effect of organic solvent, we employed a 1:1 mixture of ethanol/water, which revealed a marginal increase in the yields (Table 1, entries 8–10). However, despite the slightly lower yield, we selected water as an environmentally benign solvent. Temperature also played a vital role on the formation of the product. No product was detected at room temperature (rt), however, the yields gradually increased with increasing temperatures up to 100 °C (Table 1, entries 1–5).

**Table 1:** Effect of various reaction parameters.^a^

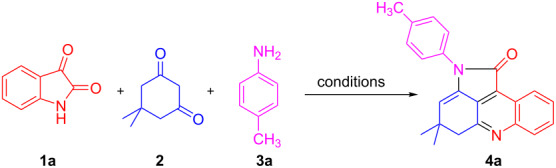					

Entry	Catalyst	Solvent	Temp. (°C)	Time, h	Yield^b^ (%)

1	BATA-MC	H_2_O	rt^c^	72	NF
2	BATA-MC	H_2_O	80	24	70
3	BATA-MC	H_2_O	80	48	72
4	BATA-MC	H_2_O	100	6	78
5	**BATA-MC**	**H_2_O**	**100**	**8**	**88**
6	BATA-MC	H_2_O	100	12	89
7	BATA-MC	H_2_O	100	24	89
8	BATA-MC	EtOH/H_2_O 1:1	80	8	85
9	BATA-MC	EtOH/H_2_O 1:1	90	8	91
10	BATA-MC	EtOH/H_2_O 1:1	90	12	92
11	–	H_2_O	100	48	NF
12	–	EtOH/H_2_O 1:1	90	48	trace
13	–	PEG/H_2_O 1:4	100	48	12
14	–	PEG/H_2_O 1:3	100	48	16

^a^Reaction conditions: a mixture of 1.0 equiv of each of dimedone (1 mmol), isatin (1 mmol), and *p*-toluidine (1 mmol) was heated with stirring in the presence of the catalyst (10 mol %) in 3 mL of solvent. ^b^Isolated yield. NF= not found.

To investigate the effectiveness of BATA-MC, the reaction was carried out in absence of the catalyst. The product **4a** did not form in water even at 100 °C (Table 1, entry 11). Only a trace amount of the product was detected when the reaction was carried out in ethanol/water 1:1 (Table 1, entry 12). Even when repeating the reaction in the presence of the phase-transfer catalyst, poly(ethylene glycol) (PEG) no mentionable yield of product was observed (Table 1, entries 13 and 14).

**Figure 3:**
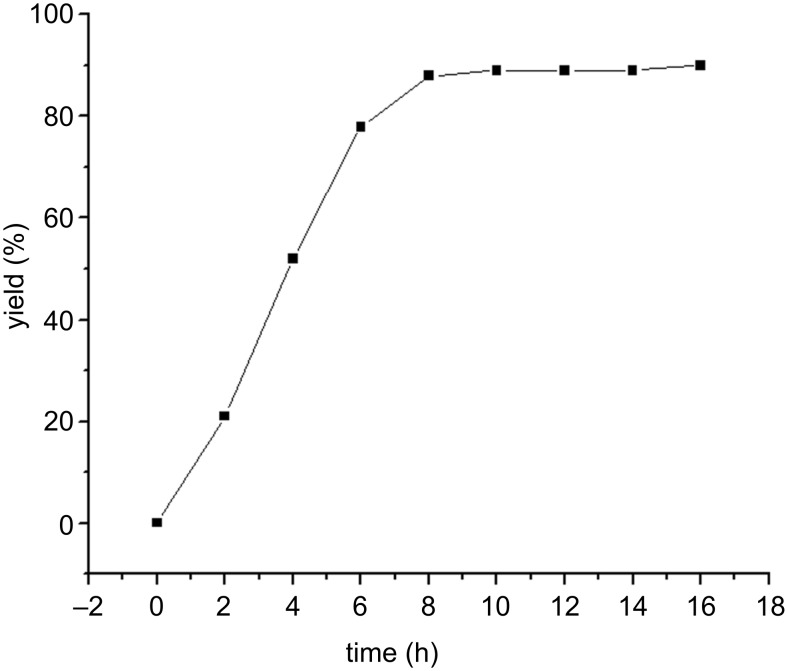
Dependence of the yield of compound **4a** on the reaction time using BATA-MC.

The subsequent screening of the kinetics of the reaction for the optimum time is shown in Figure 3. The best result was obtained at approximate 8 h and longer reaction times (up to 18 h), maintaining all reaction conditions unchanged, did not improve the yield. Thus, we converge that the BATA-MC as a nanoreactor provides excellent results (88% yield) at 100 °C over 8 h in aqueous medium (Table 1, entry 5).

### Catalyst loading

**Figure 4:**
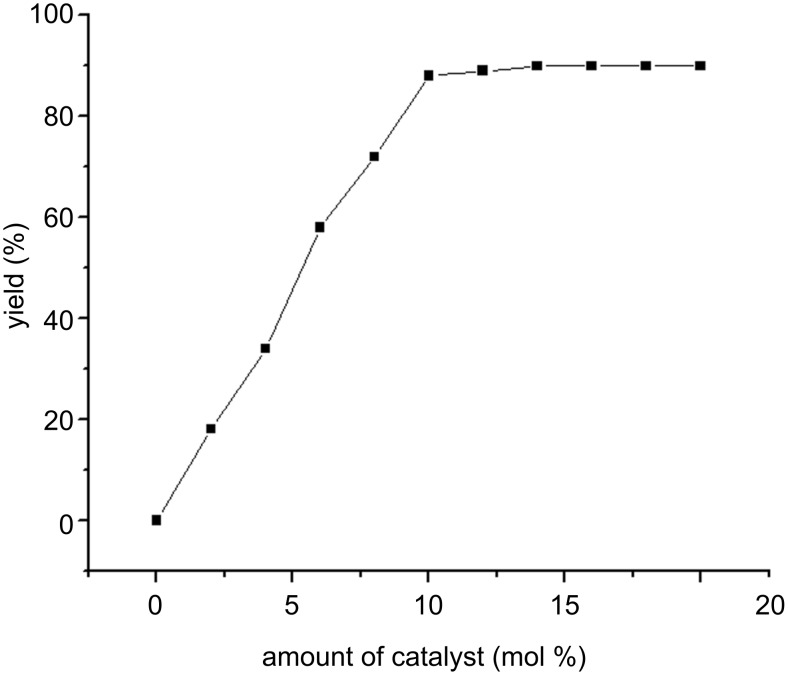
Yields of product **4a** at different catalyst loading.

To identify the optimum amount of the catalyst, the synthesis of **4a** was inspected with different amounts of BATA-MC in H_2_O at 100 °C. A consistent increase in the yield of **4a** is observed with increasing the amount of the catalyst up to 10 mol % (Figure 4). Higher catalyst concentrations had no beneficial effect on the product yield. So, we selected 10 mol % BATA-MC loading as the minimum effective amount for further reactions.

### Substrate scope

#### Synthesis of 4,5-dihydropyrrolo[2,3,4-*kl*]acridinones

**Scheme 1:**
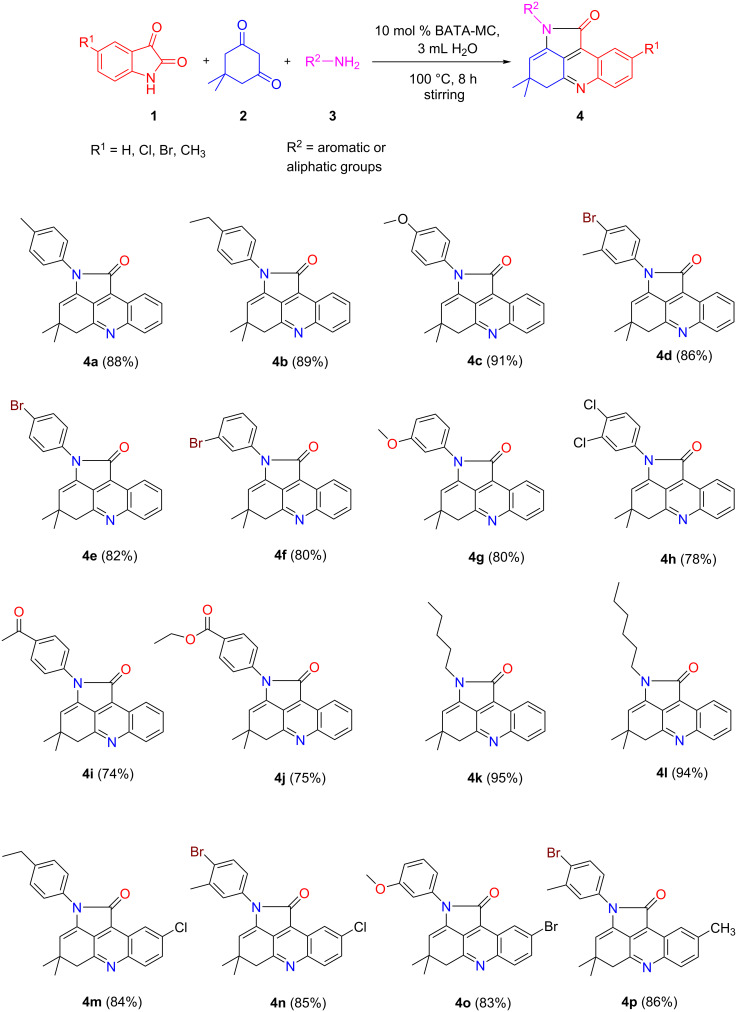
BATA-MC-catalyzed synthesis of 4,5-dihydropyrrolo[2,3,4-*kl*]acridinones.

After acquiring the optimized reaction conditions, we next turned our attention to the reaction scope. A series of investigations was performed to prepare various types of pyrroloacridines (Scheme 1). We first scrutinized a variety of anilines having different types of electron-donating or electron-withdrawing groups which showed good to excellent yields (74–91%) of the expected products **4a**–**j**. The presence of electron-donating groups showed the best results and electron-withdrawing groups resulted in slightly lower yields.

Of note, aliphatic amines showed outstanding results affording 4,5-dihydropyrrolo[2,3,4-*kl*]acridinones **4k**,**l** in high yields. Also good yields (83–86%) of products **4m**–**p** were obtained when various substituted isatins with electron-withdrawing as well as electron-donating groups were engaged in this transformation.

#### Synthesis of pyrrolo[2,3,4-*kl*]acridinones

**Scheme 2:**
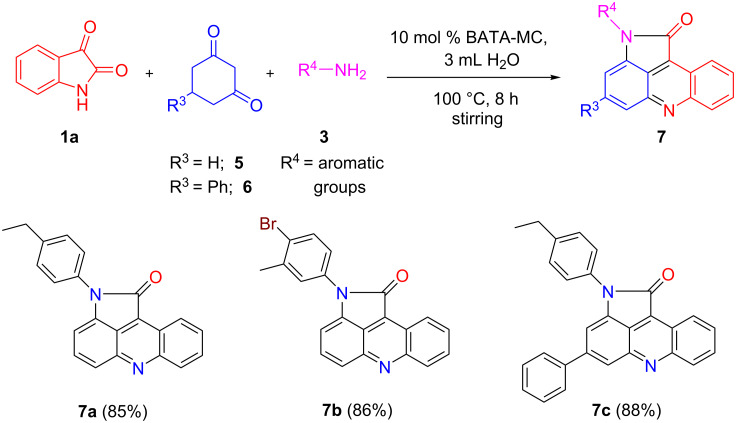
BATA-MC-catalyzed synthesis of pyrrolo[2,3,4-*kl*]acridinone derivatives.

After the successful synthesis of the 4,5-dihydropyrrolo derivatives, we were interested to synthesize fully aromatic products by using C5–H containing cyclohexane-1,3-dione derivatives in place of dimedone. Thus, using cyclohexane-1,3-dione (**5**) or 5-phenylcyclohexane-1,3-dione (**6**) allowed the isolation of novel substituted pyrrolo[2,3,4-*kl*]acridinones in high yields and illustrated the adaptability of this protocol (Scheme 2). Thus, under the above optimized conditions, the corresponding fully oxidized products were obtained through aerial oxidation or dehydrogenation of 4,5-dihydropyrrolo[2,3,4-*kl*]acridinones driven by aromatic stabilization. Consequently, the presented BATA-MC-catalyzed protocol demonstrates a general applicability furnishing a variety of multisubstituted acridines.

#### Confirmation of structures

**Figure 5:**
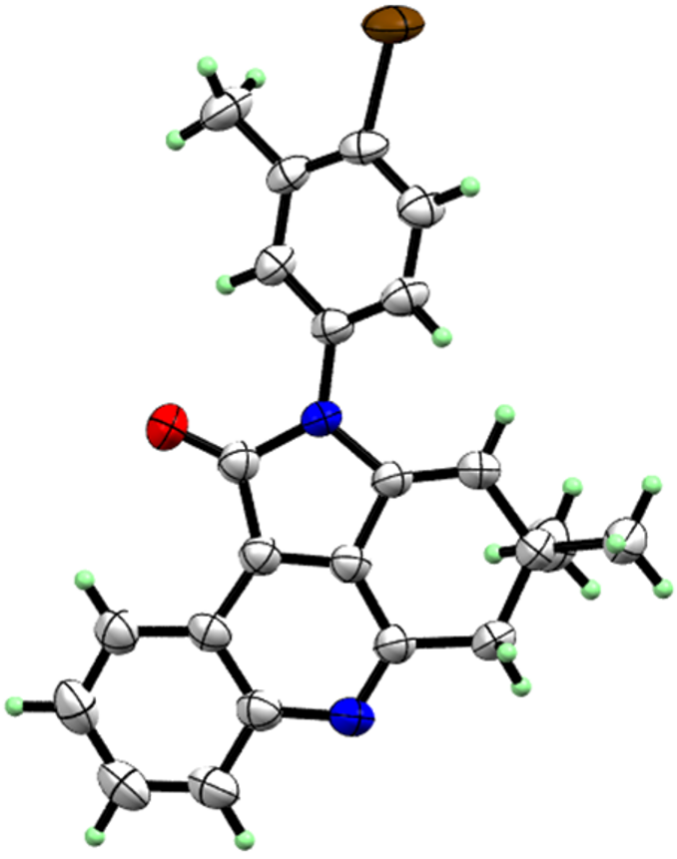
X-ray single crystal structure of **4d** (CCDC 1898008).

All products (4,5-dihydropyrrolo[2,3,4-*kl*]acridinones **4a**–**p** and pyrrolo[2,3,4-kl]acridinones **7a**–**c**) were characterized by using different spectroscopic techniques, ^1^H NMR, ^13^C NMR, IR and HRMS. In addition, one of the compounds, **4d**, was also examined by single crystal X-ray structure analysis (Figure 5).

#### Mechanistic pathway

**Scheme 3:**
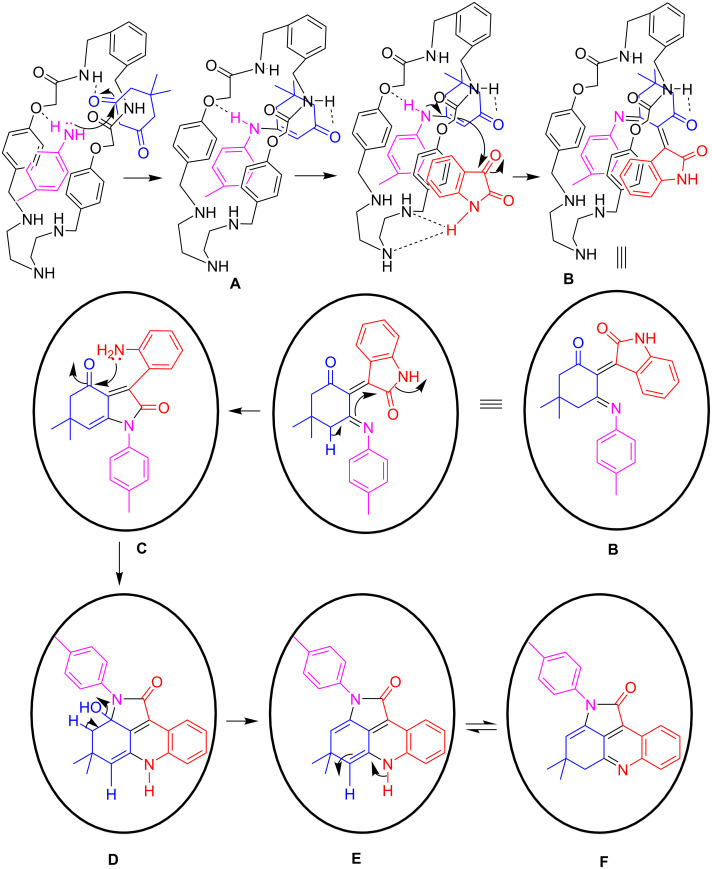
Probable mechanism illustrated for the synthesis of **4a** using BATA-MC. For the sake of simplicity, we denoted BATA-MC as the cycle in some steps.

A probable mechanism is portrayed in Scheme 3 including the role of the BATA-MC nanoreactor. The nanoreactor is a molecular assembly of BATA-MC single molecules which may provide a suitable organic environment to the starting materials by π-interaction and H-bonding, and inevitably orients them into the necessary reactive positions.

**Figure 6:**
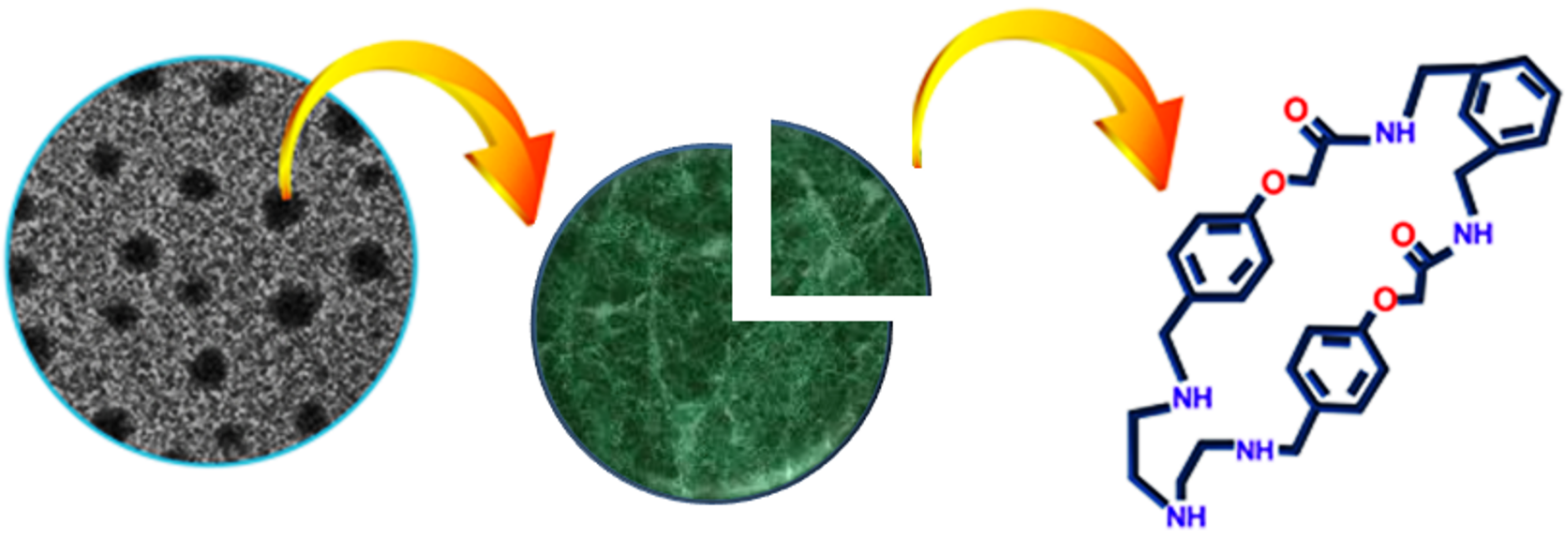
Representation of BATA-MC nanoreactor.

To represent the simple catalytic activity, we have chosen a part of the nanoreactor which is a single molecule as catalyst in the earliest steps of the mechanistic pathway (Figure 6).

This multicomponent reaction is expected to comprise a series of condensations and subsequent ring-closure cascade reactions (Scheme 3). To demonstrate the mechanistic pathway, dimedone (**2**), isatin (**1a**) and *p*-toluidine (**3a**) were selected as model substrates. Firstly, dimedone and *p*-toluidine form enaminoketone **A** through a condensation reaction and BATA-MC provides the environment for this condensation in water. To investigate this primary step, a control experiment was performed. One aliquot was taken from the reaction mixture after 10 minutes and analyzed by ESIMS. The observed peak at *m*/*z* 801.4 corresponding to [BATA-MC + **2** + **3a** + Na]^+^ confirmed the involvement of BATA-MC as nanoreactor (Supporting Information File 1, Figure S1). When we investigated the interaction of isatin with BATA-MC, we observed an ESIMS peak at *m*/*z* 679.32 corresponding to [BATA-MC + **1a** + H]^+^ (Supporting Information File 1, Figure S2) which supported the association of the macrocycle with isatin. Hence, further condensation between enaminoketone and isatin provides the intermediate **B**. The isatin moiety also has π-stacking sites along with H-bonding donor/acceptor site. So, it may also be associated to the nanoreactor to approach the enaminoketone. Then, consecutive cyclization and ring opening of isatin (translactamization) affords intermediate **C** which undergoes cyclocondensation to furnish the final product **F**.

### Recyclability of the BATA-MC catalyst

**Figure 7:**
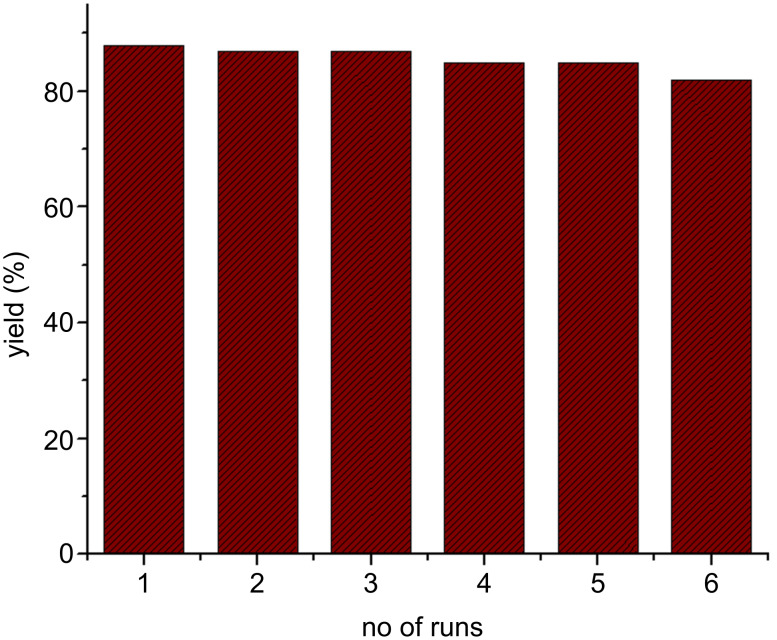
The reusability of the nanoreactor for the synthesis of **4a**.

The BATA-MC catalyst was recovered by column chromatography and its recyclability was investigated in the synthesis of **4a** to demonstrate the eco-friendly nature of the catalyst. The catalytic activity remained nearly unchanged up to five cycles as shown in Figure 7. Therefore, BATA-MC has the prospective of reworking at least five times. SEM and TEM images of the catalyst’s nanoranged dispersive particles after five cycles are given in Supporting Information File 1 (Figure S4) which confirm the range of particle sizes of 70–110 nm even after five cycles*.*

## Conclusion

In summary, a multifunctional heteroditopic macrocycle is established as an efficient nanoreactor for organic transformations in water. Using this macrocycle, a library of biologically interesting highly substituted pyrroloacridines has been synthesized with high yields from easily available starting materials in water within 8 h. Following this green methodology, we successfully synthesized 4,5-dihydropyrroloacridinones and fully aromatized pyrrolo analogues in the eco-friendly solvent water with BATA-MC as a recyclable catalyst. Moreover, some of the synthesized acridines may find medicinal applications in future.

## Experimental

### General procedure for the synthesis of pyrrolo[2,3,4-*kl*]acridinone derivatives

First 53.1 mg (10 mol %) of BATA-MC was dissolved in 3 mL of H_2_O by ultrasonication for five minutes. Then, 5,5-dimethylcyclohexane-1,3-dione (140 mg, 1 mmol), aromatic or aliphatic amine (1 mmol) and isatin (147 mg, 1 mmol) were added to the solution. The reaction mixture was refluxed at 100 °C with stirring for 8 h. The progress of the reaction was monitored by TLC. After completion of the reaction, the organic part was extracted with chloroform and then subjected to column chromatography using 60–120 mesh silica gel and petroleum ether/ethyl acetate mixture as eluent. We got pure products at 3–4% ethyl acetate in petroleum ether and the pure catalyst using 5% methanol in dichloromethane.

## Supporting Information

File 1 Analytical data and copies of ^1^H, ^13^C NMR and MS spectra.
