# Supplementary material for: A heteroditopic macrocycle as organocatalytic nanoreactor for pyrroloacridinone synthesis in water
Source: Beilstein J Org Chem. 2019 Jul 8;15:1505–14. doi: 10.3762/bjoc.15.152 (PMC6632221; doi:10.3762/bjoc.15.152)
Supplement: File 1 — Analytical data and copies of 1H, 13C NMR and MS spectra. [file Beilstein_J_Org_Chem-15-1505-s001.pdf]

BEILSTEIN JOURNAL OF ORGANIC CHEMISTRY

Supporting Information

for

A heteroditopic macrocycle as organocatalytic nanoreactor for pyrroloacridinone synthesis in water

Piyali Sarkar, Sayan Sarkar and Pradyut Ghosh

Beilstein J. Org. Chem. **2019,** *15,* 1505–1514. doi:10.3762/bjoc.15.152

Analytical data and copies of ^{1}H, ^{13}C NMR and MS spectra

License and Terms: This is a supporting information file under the terms of the Creative Commons Attribution License (http://creativecommons.org/licenses/by/4.0). Please note that the reuse, redistribution and reproduction in particular requires that the authors and source are credited.
The license is subject to the *Beilstein Journal of Organic Chemistry* terms and conditions: (https://www.beilstein-journals.org/bjoc)

Table of Contents

General....................S1

Synthesis of BATA-MC (Scheme S1)....................S2

ESI-MS of [BATA-MC + **2** + **3a** + Na^+] (Figure S1)....................S2

ESI-MS of [BATA-MC + **1** + H^+] (Figure S2)....................S3

Stability of macrocycle at high temperature up to 100 ºC and stack plot of temperature dependent ^{1}H NMR of the macrocycle (Figure S3)....................S3

SEM image and TEM image of nano-ranged dispersive particles of macrocycle

after five cycles (Figure S4)....................S4

Spectral and analytical data of compounds **4a–p** and **7a–c** S4

^{1}H and ^{13}C spectra of all the products (Figure S5 to Figure S42)....................S11

References....................S30

General:

All starting materials were purchased from commercial sources such as Sigma Aldrich, Merck, Spectrochem and Alfa Aesar and are used as received without further purification. Melting points of all compounds were determined on a Labtronics digital auto melting/boiling point apparatus. Electrospray ionisation mass spectrometry (ESIMS) experiments were carried out on a Water's QtoF Model YA 263 spectrometer in the positive ion ESI mode. IR data were recorded on a SHIMADZU FTIR-8400S Infrared spectrophotometer. All NMR experiments were obtained on 400 MHz Bruker DPX. TEM images were captured using a JEOL JEM2010/11 (for high-resolution (HR)TEM) instrument by using 300 mesh carbon-coated copper TEM grid. SEM images were obtained with a JEOL JMS-6700F field-emission scanning electron microscope.

Single crystals of compound **4d** suitable for X-ray analysis were obtained by slow evaporation of an ethyl acetate solution of the compound. Single crystal X-ray diffraction data were collected using a Bruker APEX II, CCD area detector, MoKα, λ = 0.7107 Å). Data collection,

data reduction, structure solution and refinement were carried out using the software package of the corresponding diffractometer (SMART APEX-II).

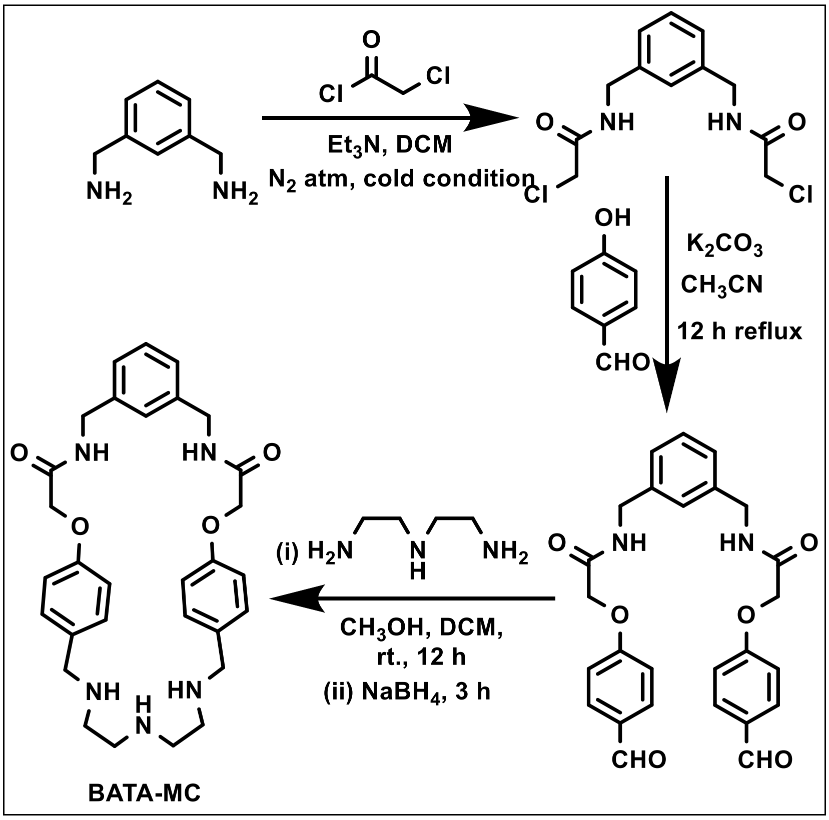

Scheme S1: Synthesis of BATA-MC

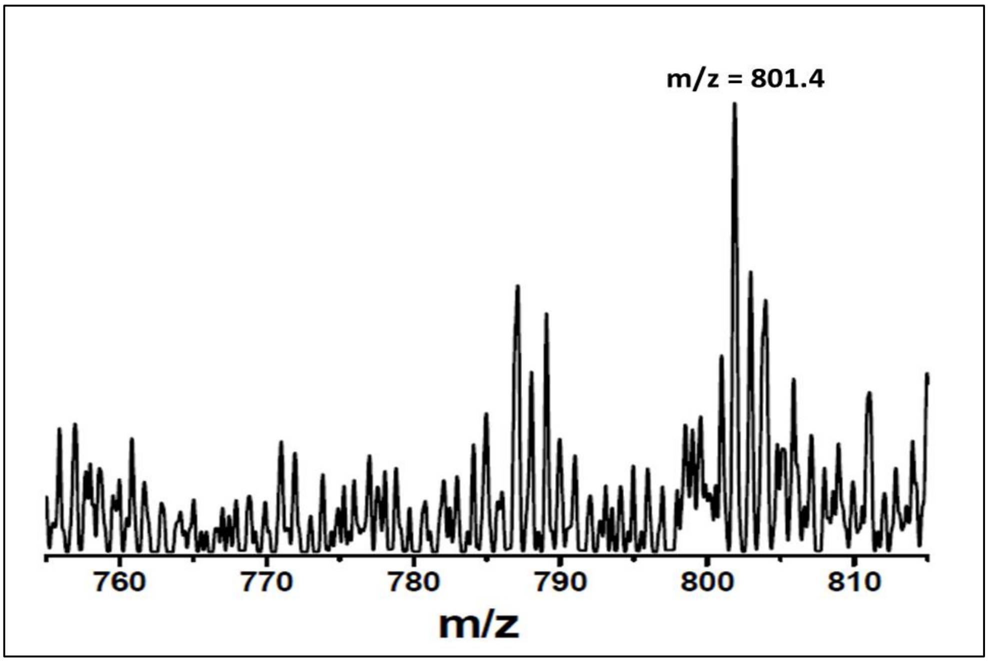

Figure S1: ESIMS of [BATA-MC + **2** + **3a** + Na^+]

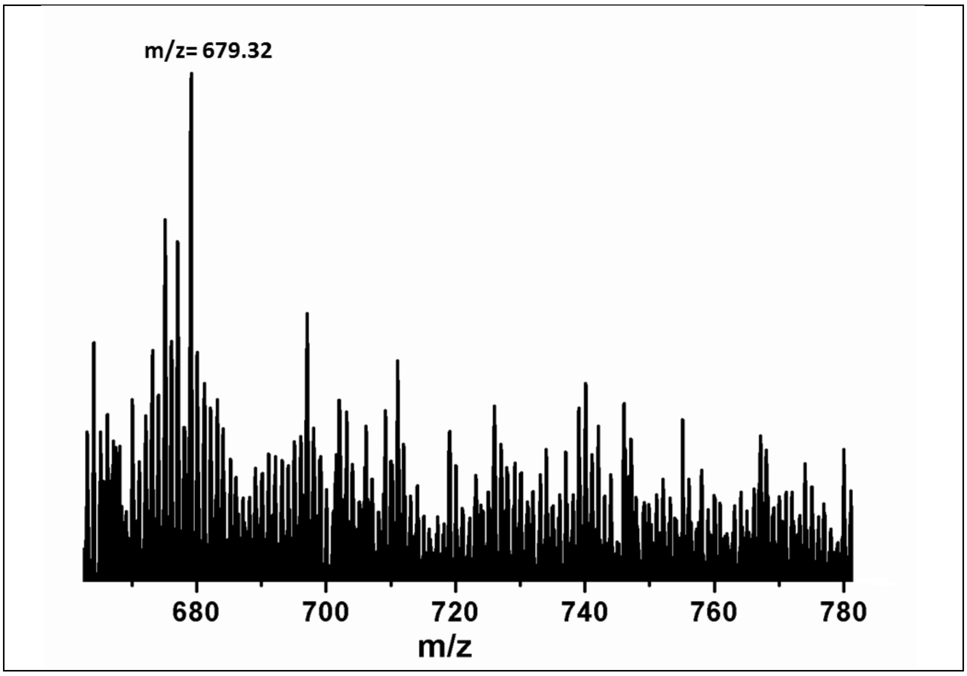

Figure S2: ESIMS peak for [BATA-MC + **1a** + H^+]

The stability of the macrocycle was tested at high temperature up to 100 ºC by recording the ^{1}H NMR spectrum at 100 ºC. The spectrum is given below. The high temperature spectrum exactly matches that recorded at room temperature, which reveals the stability of the macrocycle at high temperature.

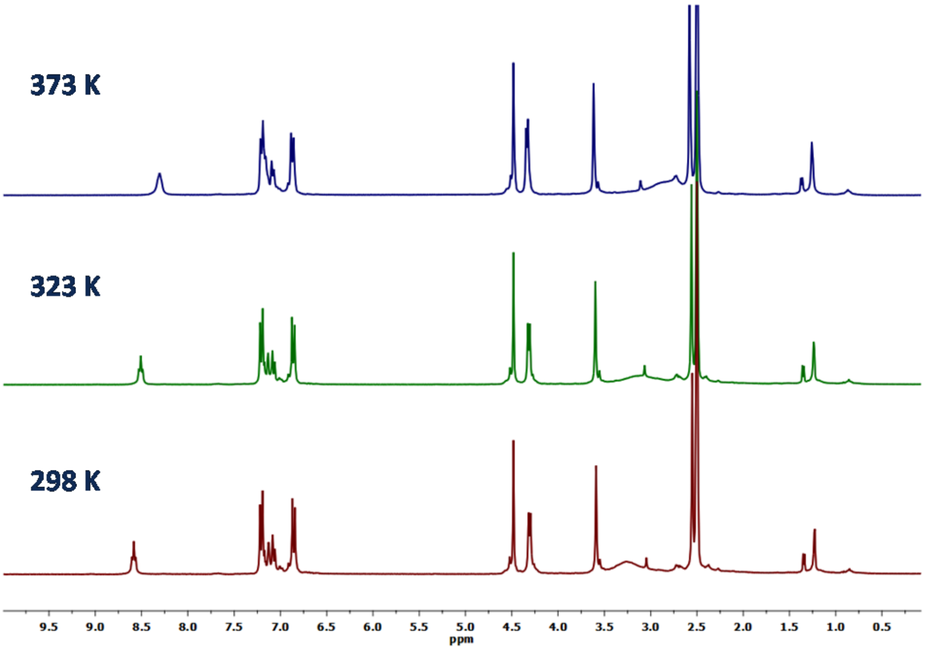

Figure S3: Stack plot of temperature-dependent ^{1}H NMR of the macrocycle.

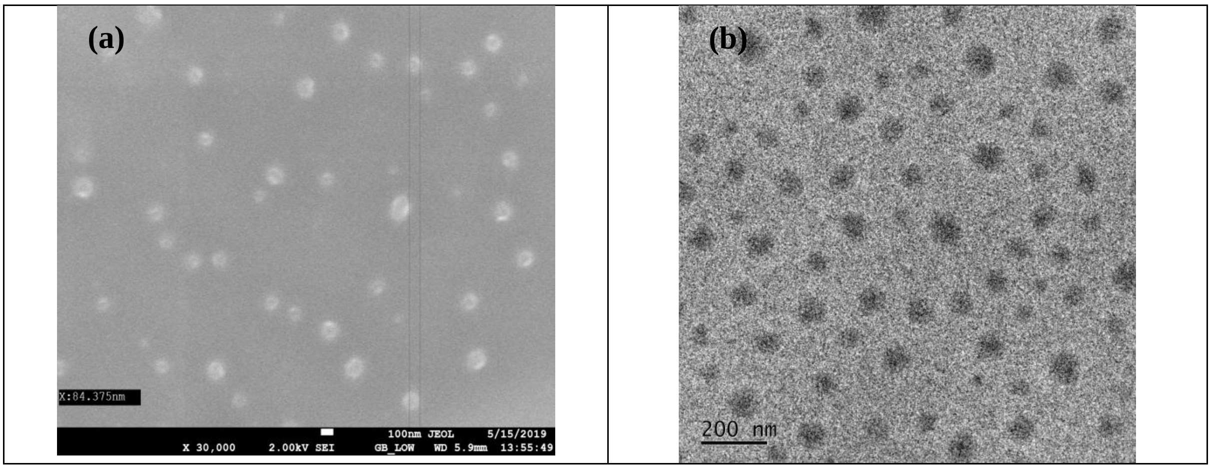

Figure S4: (a) SEM image and (b) TEM image of nano-ranged dispersive particles of the macrocycle after five cycles.

Spectral and analytical data of the products:

4,4-Dimethyl-2-*p*-tolyl-4,5-dihydropyrrolo[2,3,4-*kl*]acridin-1(2*H*)-one (4a)[1]:

Yellow solid (88%), mp. 216 ºC (EtOAc); IR (KBr, υ cm^{-1}): 2956, 1706, 1483, 1344, 1078, 779, 684; ^{1}H NMR (400 MHz, $CDCl_3$): δ (ppm) 1.32 (s, 6H), 2.43 (s, 3H), 3.21 (s, 2H), 5.59 (s, 1H), 7.36 (dd, 4H, J=10Hz, 8.4Hz), 7.64 (t, 1H, J=7.8Hz), 7.74 (t, 1H, J=8.0Hz), 8.17 (d, 1H, J=8.4Hz), 8.72 (d, 1H, J=7.6Hz); ^{13}C NMR (100 MHz, $CDCl_3$): 21.3, 31.0, 37.2, 44.3, 118.3, 122.8, 124.4, 125.3, 126.4, 126.6, 127.9, 129.4, 129.6, 130.1, 132.2, 133.7, 137.6, 149.7, 154.6, 166.8; HRMS (ESI-TOF) *m/z*: $[M + H]^+$ Calcd for [$C_{25}H_{23}N_2O_3$]: 341.1641, Found 341.1645.

2-(4-Ethylphenyl)-4,4-dimethyl-4,5-dihydropyrrolo[2,3,4-*kl*]acridin-1(2*H*)-one (4b): Yellow solid (89%), mp. 204-206 ºC (EtOAc) ; IR (KBr, γ cm^{-1}): 2967, 1705, 1581, 1492, 1222, 779; ^{1}H NMR (400 MHz, $CDCl_3$): δ (ppm) 1.28-1.33 (m, 9H), 2.74 (q, 2H, J=8.0Hz), 3.28 (s, 2H), 5.64 (s, 1H), 7.36-7.42 (m, 4H), 7.69 (t, 1H, J=7.2Hz), 7.79 (t, 1H, J=8.0Hz), 8.26-8.27 (m, 1H), 8.75 (d, 1H, J=8.0Hz); ^{13}C NMR (100 MHz, $CDCl_3$): 15.6, 28.7, 31.0, 37.2, 44.0, 118.6, 122.8, 124.5, 125.6, 126.4, 126.6, 128.1, 129.0,

129.1, 129.9, 132.3, 133.6, 143.9, 149.2, 154.6, 166.8; HRMS (ESI-TOF) *m/z*: [M + H]$^+$ Calcd for [$C_{24}H_{23}N_2O$]: 355.1797, Found 355.1794.

2-(4-Methoxyphenyl)-4,4-dimethyl-4,5-dihydropyrrolo[2,3,4-*kl*]acridin-1(2*H*)-one (4c)[2]: Yellow solid (91%), mp. 190-192 ºC (EtOAc); IR (KBr, υ cm^{-1}): 2934, 1709, 1605, 1456, 1224, 779; ^{1}H NMR (400 MHz, $CDCl_3$): δ 1.33 (s, 6H), 3.31 (s, 2H), 3.88 (s, 3H), 5.59 (s, 1H), 7.06 (d, 2H, J=8.8Hz), 7.40 (d, 2H, J=8.8Hz), 7.70 (t, 1H, J=7.4Hz), 7.80 (t, 1H, J=7.6Hz), 8.30 (d, 1H, J=7.6Hz), 8.75 (d, 1H, J=8.0Hz); ^{13}C NMR (100 MHz, $CDCl_3$): 31.1, 37.3, 38.9, 55.7, 114.9, 118.7, 122.9, 124.5, 126.6, 127.3, 128.0, 128.4, 129.0, 130.4, 131.0, 132.6, 133.6, 133.7, 154.4, 159.2; HRMS (ESI-TOF) *m/z*: [M + H]$^+$ Calcd for [$C_{23}H_{21}N_2O_2$]: 357.1563, Found 357.1560.

2-(4-Bromo-3-methylphenyl)-4,4-dimethyl-4,5-dihydropyrrolo[2,3,4-*kl*]acridin-1(2*H*)-one (4d): Yellow solid (86%), mp. 188 ºC (EtOAc); IR (KBr, υ cm^{-1}): 2928, 1705, 1483, 1346, 1074, 781; ^{1}H NMR (400 MHz, $CDCl_3$): δ (ppm) 1.33 (s, 6H), 2.49 (s, 3H), 3.24 (s, 2H), 5.61 (s, 1H), 7.18 (d, 1H, J=8.4Hz), 7.42 (s, 1H), 7.66-7.70 (m, 2H), 7.78 (t, 1H, J=7.8Hz), 8.21 (d, 1H, J=8.0Hz), 8.72 (d, 1H, J=8.0Hz); ^{13}C NMR (100 MHz, $CDCl_3$): 23.3, 31.0, 37.3, 44.0, 118.7, 122.7, 124.0, 124.4, 125.3, 126.6, 128.2, 128.8, 129.2, 130.1, 133.2, 133.4, 133.9, 139.6, 149.2, 154.5, 166.6; HRMS (ESI-TOF) *m/z*: [M + H]$^+$ Calcd for [$C_{23}H_{20}BrN_2O$]: 419.0781, Found 419.0779.

2-(4-Bromophenyl)-4,4-dimethyl-4,5-dihydropyrrolo[2,3,4-*kl*]acridin-1(2*H*)-one (4e)[3]: Yellow solid (82%), mp. 202 ºC (EtOAc); IR (KBr, υ cm^{-1}): 2958, 1704, 1481, 1345, 1082, 778; ^{1}H NMR (400 MHz, $CDCl_3$): δ (ppm) 1.33 (s, 6H), 3.22 (s, 2H), 5.62 (s,1H), 7.40 (d, 2H, J=8.4Hz), 7.66-7.68 (m, 3H), 7.76 (t, 1H, J=8.0Hz), 8.18 (d, 1H, J=8.4Hz), 8.70 (d, 1H, J=8.0Hz); ^{13}C NMR (100 MHz, $CDCl_3$): 31.0, 37.3, 44.0, 118.7, 121.3, 122.7, 124.3, 125.2, 126.6, 128.0, 128.2, 129.3, 130.0, 132.7, 133.1, 133.9,

149.4, 154.5, 166.5; HRMS (ESI-TOF) *m/z*: $[M + H]^+$ Calcd for $[C_{22}H_{18}BrN_2O]$: 405.0625, Found 405.0626.

2-(3-Bromophenyl)-4,4-dimethyl-4,5-dihydropyrrolo[2,3,4-*kl*]acridin-1(2*H*)-one (4f): Yellow solid (80%), mp. 183 ºC (EtOAc); IR (KBr, υ cm^{-1}): 2957, 1705, 1483, 1339, 1078, 779, 685; ^1H NMR (400 MHz, $CDCl_3$): δ (ppm) 1.33 (s, 6H), 3.21 (s, 2H), 5.64 (s, 1H), 7.38-7.46 (m, 2H), 7.53 (d, 1H, 7.6Hz), 7.64-7.69 (m, 2H), 7.75 (t, 1H, 7.8Hz), 8.17 (d, 1H, 8.4Hz), 8.69 (d, 1H, 8.0Hz); ^{13}C NMR (100 MHz, $CDCl_3$): 30.9, 37.2, 44.2, 118.6, 122.6, 122.9, 124.3, 124.9, 125.1, 126.6, 128.1, 129.5, 129.5, 129.9, 130.7, 130.7, 133.1, 136.2, 149.7, 154.6, 166.6; HRMS (ESI-TOF) *m/z*: $[M + H]^+$ Calcd for $[C_{22}H_{18}BrN_2O]$: 405.0625, Found 405.0623.

2-(3-Methoxyphenyl)-4,4-dimethyl-4,5-dihydropyrrolo[2,3,4-*kl*]acridin-1(2*H*)-one (4g)[2]: Reddish yellow solid (80%), mp. 206 ºC (EtOAc); IR (KBr, υ cm^{-1}): 2924, 1709, 1603, 1460, 1222, 777; ^1H NMR (400 MHz, $CDCl_3$): δ (ppm) 1.33 (s, 6H), 3.24 (s, 2H), 3.87 (s, 3H), 5.67 (s, 1H), 6.95-6.97 (m, 1H), 7.07-7.08 (m, 2H), 7.45 (t, 1H, J=8.4Hz), 7.68 (t, 1H, J=7.4Hz), 7.77 (t, 1H, J=7.2Hz), 8.21 (d, 1H, J=8.4Hz), 8.74 (d, 1H, J=8.0Hz); ^{13}C NMR (100 MHz, $CDCl_3$): 31.0, 37.3, 44.0, 55.7, 112.5, 113.4, 118.7, 118.9, 122.8, 124.4, 125.4, 126.6, 128.2, 129.2, 130.0, 130.2, 133.4, 135.9, 149.2, 154.6, 160.5, 166.6; HRMS (ESI-TOF) *m/z*: $[M + H]^+$ Calcd for $[C_{23}H_{21}N_2O_2]$: 357.1563, Found 357.1567.

2-(3,4-Dichlorophenyl)-4,4-dimethyl-4,5-dihydropyrrolo[2,3,4-*kl*]acridin-1(2*H*)-one (4h): Yellow solid (78%), mp. 238 ºC (EtOAc): IR (KBr, υ cm^{-1}): 2954, 1705, 1589, 1481, 1344, 1078, 779, 684; ^1H NMR (400 MHz, $CDCl_3$): δ (ppm) 1.34 (s, 6H), 3.23 (s, 2H), 5.64 (s, 1H), 7.39 (dd, 1H, J=8.8Hz, 2.4Hz), 7.62 (d, 1H, J=8.8Hz), 7.66-7.70 (m, 2H), 7.78 (t, 1H, J=7.8Hz), 8.20 (d, 1H, J=8.4Hz), 8.70 (d, 1H, J=8.0Hz); ^{13}C NMR (100 MHz, $CDCl_3$): 31.0, 37.3, 44.0, 118.8, 122.6, 124.3, 125.0, 125.6, 126.6, 127.8,

128.3, 129.4, 130.1, 131.2, 131.7, 132.8, 133.5, 134.3, 149.5, 154.5, 166.4; HRMS (ESI-TOF) *m/z*: $[M + H]^+$ Calcd for $[C_{22}H_{17}Cl_2N_2O]$: 395.0703, Found 395.0700.

2-(4-Acetylphenyl)-4,4-dimethyl-4,5-dihydropyrrolo[2,3,4-*kl*]acridin-1(2*H*)-one (4i): Reddish yellow solid (74%), mp. 212 ºC (EtOAc); IR (KBr, υ cm^{-1}): 2924, 1709, 1682, 1599, 1462, 1267, 775; ^{1}H NMR (400 MHz, $CDCl_3$): δ (ppm) 1.34 (s, 6H), 2.67 (s, 3H), 3.25 (s, 2H), 5.72 (s, 1H), 7.65-7.71 (m, 3H), 7.78 (t, 1H, J=7.6Hz), 8.14 (d, 2H, J=8.8Hz), 8.21 (d, 1H, J=8.4Hz), 8.72 (d, 1H, J=8.0Hz); ^{13}C NMR (100 MHz, $CDCl_3$): 26.8, 30.9, 37.3, 43.9, 119.2, 122.6, 124.3, 125.1, 126.0, 126.7, 128.3, 129.3, 129.7, 130.1, 132.7, 135.7, 139.2, 149.3, 154.6, 166.4, 197.1; HRMS (ESI-TOF) *m/z*: $[M + H]^+$ Calcd for $[C_{24}H_{21}N_2O_2]$: 369.1563, Found 369.1561.

Ethyl 4-(4,4-dimethyl-1-oxo-4,5-dihydropyrrolo[2,3,4-*kl*]acridin-2(1*H*)yl)benzoate (4j): Yellow solid (75%), mp. 226 ºC (EtOAc); IR (KBr, υ cm^{-1}): 2924, 1717, 1605, 1271, 1103, 673; ^{1}H NMR (400 MHz, $CDCl_3$): ^{1}H NMR (400 MHz, $CDCl_3$): δ (ppm) 1.34 (s, 6H), 1.43 (t, 3H, J=7.2Hz), 3.25 (s, 2H), 4.42 (q, 2H, J=7.2Hz), 5.70 (s, 1H), 7.63 (d, 2H, J=8.4Hz), 7.69 (t, 1H, J=7.4Hz), 7.78 (t, 1H, J=7.2Hz), 8.20-8.24 (m, 3H), 8.73 (d, 1H, J=8.0Hz); ^{13}C NMR (100 MHz, $CDCl_3$): 14.5, 31.0, 37.3, 44.0, 61.4, 119.1, 122.7, 124.4, 125.2, 125.8, 126.7, 128.3, 129.3, 130.1, 130.9, 132.8, 139.0, 154.6, 166.0, 166.4; HRMS (ESI-TOF) *m/z*: $[M + H]^+$ Calcd for $[C_{25}H_{23}N_2O_3]$: 399.1719, Found 399.1723.

4,4-Dimethyl-2-pentyl-4,5-dihydropyrrolo[2,3,4-*kl*]acridin-1(2*H*)-one (4k): Yellow solid (95%), mp. 138 ºC (EtOAc); IR (KBr, υ cm^{-1}): 2944, 1703, 1515, 1231, 1054, 778; ^{1}H NMR (400 MHz, $CDCl_3$): δ (ppm) 0.87 (t, 3H, J=6.6Hz), 1.30-1.35 (m, 10H), 1.68-1.73 (m, 2H), 3.13 (s, 2H), 3.75 (t, 2H, J=7.2Hz), 5.49 (s, 1H), 7.58 (t, 1H, J=7.4Hz), 7.67 (t, 1H, J=7.0Hz), 8.10 (d, 1H, J=8.4Hz), 8.63 (d, 1H, J=7.6Hz); ^{13}C NMR (100 MHz, $CDCl_3$): 14.0, 22.4, 28.8, 29.2, 31.1, 37.1, 40.2, 44.2, 116.7, 122.7, 124.2, 125.8,

126.4, 127.6, 129.3, 129.4, 133.3, 149.6, 154.3, 167.5; HRMS (ESI-TOF) *m/z*: [M + H]+ Calcd for [$C_{21}H_{25}N_2O$]: 321.1953, Found 321.1952.

2-Hexyl-4,4-dimethyl-4,5-dihydropyrrolo[2,3,4-*kl*]acridin-1(2*H*)-one (4l): Yellow solid (94%), mp. 148-150 ºC (EtOAc); IR (KBr, υ cm^{-1}): 2939, 1706, 1497, 1213, 1047, 783; ^{1}H NMR (400 MHz, $CDCl_3$): δ (ppm) 0.86 (t, 3H, J=7.0Hz), 1.32 (brs, 12H), 1.67-1.74 (m, 2H), 3.14 (s, 2H), 3.77 (t, 2H, J=7.4Hz), 5.50 (s, 1H), 7.60 (t, 1H, J=7.4Hz), 7.70 (t, 1H, J=7.0Hz), 8.12 (d, 1H, J=8.4Hz), 8.65 (d, 1H, J=8.0Hz); ^{13}C NMR (100 MHz, $CDCl_3$): 14.1, 22.6, 26.7, 29.2, 31.1, 31.6, 37.1, 40.3, 44.2, 116.7, 122.7, 124.3, 125.9, 126.5, 127.6, 129.3, 129.4, 133.4, 149.6, 154.4, 167.5; HRMS (ESI-TOF) *m/z*: [M + H]+ Calcd for [$C_{22}H_{27}N_2O$]: 335.2109, Found 335.2111.

9-Chloro-2-(4-ethylphenyl)-4,4-dimethyl-4,5-dihydropyrrolo[2,3,4-*kl*]acridin-1(2*H*)-one (4m): Yellow solid (84%), mp. 198 ºC (EtOAc)**;** IR (KBr, υ cm^{-1}): 2935, 1708, 1509, 1305, 786; ^{1}H NMR (400 MHz, $CDCl_3$): δ (ppm) 1.30-1.32 (m, 9H), 2.73 (q, 2H, J=7.5Hz), 3.19 (s, 2H), 5.65 (s, 1H), 7.35-7.40 (m, 4H), 7.67 (d, 1H, J=8.8Hz), 8.08 (d, 1H, J=9.2Hz), 8.69 (s, 1H); ^{13}C NMR (100 MHz, $CDCl_3$): 15.6, 28.7, 31.0, 37.3, 44.1, 119.2, 123.4, 123.4, 124.5, 126.0, 126.4, 127.1, 129.0, 129.2, 130.5, 130.8, 132.2, 133.4, 134.0, 144.0, 147.9, 155.0, 166.3; HRMS (ESI-TOF) *m/z*: [M + H]+ Calcd for [$C_{24}H_{22}ClN_2O$]: 389.1406, Found 389.1403.

2-(4-Bromo-3-methylphenyl)-9-chloro-4,4-dimethyl-4,5-dihydropyrrolo[2,3,4-*kl*]acridin-1(2*H*)-one (4n): Yellow solid (85%), mp. 174-176 ºC (EtOAc); IR (KBr, υ cm^{-1}): 2942, 1705, 1603, 1456, 1231, 1042, 780; ^{1}H NMR (400 MHz, $CDCl_3$): δ (ppm) 1.32 (s, 6H), 2.48 (s, 3H), 3.20 (s, 2H), 5.63 (s, 1H), 7.16 (d, 1H, J=8.4Hz), 7.39 (s, 1H), 7.67-7.70 (m, 2H), 8.09 (d, 1H, J=9.2Hz), 8.66 (s, 1H); ^{13}C NMR (100 MHz, $CDCl_3$): 23.3, 31.0, 37.3, 44.0, 119.5, 123.2, 123.4, 124.1, 124.3, 125.2, 127.1, 128.7,

130.0, 130.7, 133.1, 133.4, 133.8, 134.3, 139.6, 147.8, 154.9, 166.1; HRMS (ESI-TOF) *m/z*: [M + H]$^+$ Calcd for [$C_{23}H_{19}BrClN_2O$]: 453.0391, Found 453.0393.

9-Bromo-2-(3-methoxyphenyl)-4,4-dimethyl-4,5-dihydropyrrolo[2,3,4-*kl*]acridin-1(2*H*)-one (4o)[2]: Reddish yellow solid (83%), mp. 175 ºC (EtOAc); IR (KBr, υ cm^{-1}): 2938, 1706, 1599, 1461, 1204, 875, 768; ^{1}H NMR (400 MHz, $CDCl_3$): δ (ppm) 1.32 (s, 6H), 3.18 (s, 2H), 3.86 (s, 3H), 5.70 (s, 1H), 6.95 (d, 1H, J=9.6Hz), 7.06 (brs, 2H), 7.44 (t, 1H, J=8.4Hz), 7.80 (d, 1H, J=11Hz), 8.00 (d, 1H, J=9.2Hz), 8.85 (s, 1H); ^{13}C NMR (100 MHz, $CDCl_3$): 31.0, 37.3, 44.1, 55.6, 112.3, 113.5, 118.6, 119.6, 122.3, 123.7, 124.1, 126.6, 127.0, 130.2, 130.9, 133.1, 135.8, 148.2, 155.1, 160.5, 166.2; HRMS (ESI-TOF) *m/z*: [M + H]$^+$ Calcd for [$C_{23}H_{20}BrN_2O_2$]: 435.0703, Found 435.0700.

2-(4-Bromo-3-methylphenyl)-4,4,9-trimethyl-4,5-dihydropyrrolo[2,3,4-*kl*]acridin-1(2*H*)-one (4p): Yellow solid (86%), mp. 184 ºC (EtOAc); IR (KBr, υ cm^{-1}): 2946, 1706, 1569, 1326, 1044, 769; ^{1}H NMR (400 MHz, $CDCl_3$): δ (ppm) 1.32 (s, 6H), 2.49 (s, 3H), 2.59 (s, 3H), 3.21 (s, 2H), 5.59 (s, 1H), 7.18 (d, 1H, J=8.4Hz), 7.41 (s, 1H), 7.59 (d, 1H, J=8.4Hz), 7.68 (d, 1H, J=8.4Hz), 8.08 (d, 1H, J=8.4Hz), 8.49 (s, 1H); ^{13}C NMR (100 MHz, $CDCl_3$): 21.9, 23.3, 31.0, 37.2, 43.8, 118.5, 122.7, 123.4, 123.9, 125.2, 126.6, 128.8, 132.2, 133.3, 134.0, 138.7, 139.5, 153.4, 166.7; HRMS (ESI-TOF) *m/z*: [M + H]$^+$ Calcd for [$C_{24}H_{22}BrN_2O$]: 433.0938, Found 433.0933.

2-(4-Ethylphenyl)pyrrolo[2,3,4-*kl*]acridin-1(2*H*)-one (7a): Yellow solid (85%), mp. 234 ºC (EtOAc); IR (KBr, υ cm^{-1}): 2934, 1705, 1601, 1224, 777; ^{1}H NMR (300 MHz, $CDCl_3$): ^{1}H NMR (400 MHz, $CDCl_3$): δ (ppm) 1.32 (t, 3H, J=7.6Hz), 2.76 (q, 2H, J=7.6Hz), 6.98 (d, 1H, J=6.8Hz), 7.41 (d, 2H, J=8.0Hz), 7.53 (d, 2H, J=8.0Hz), 7.68 (t, 1H, J=8.0Hz), 7.80 (t, 1H, J=7.6Hz), 7.87-7.94 (m, 2H), 8.45 (d, 1H, J=8.8Hz), 8.91 (d, 1H, J=8.4Hz); ^{13}C NMR (100 MHz, $CDCl_3$): 15.6, 28.8, 106.1, 119.9, 122.5, 123.2, 124.3, 125.9, 128.1, 129.2, 129.3, 130.7, 130.9, 132.4, 133.1, 140.5, 144.1, 146.4,

167.3; HRMS (ESI-TOF) *m/z*: $[M + H]^+$ Calcd for $[C_{22}H_{17}N_2O]$: 325.1328, Found 325.1326.

2-(4-Bromo-3-methylphenyl)pyrrolo[2,3,4-*kl*]acridin-1(2*H*)-one (7b): Yellow solid (86%), mp. 198 ºC (EtOAc); IR (KBr, υ cm^{-1}): 2950, 1707, 1589, 1302, 1114, 775; ^{1}H NMR (400 MHz, $CDCl_3$): δ (ppm) 2.51 (s, 3H), 6.99 (d, 1H, J=6.8Hz), 7.32-7.34 (m, 1H), 7.52 (s, 1H), 7.68-7.74 (m, 2H), 7.81 (q, 1H, J=8.0Hz), 7.91-7.96 (m, 2H), 8.47 (d, 1H, J=8.4Hz), 8.89 (d, 1H, J=8.0Hz); ^{13}C NMR (100 MHz, $CDCl_3$): 23.3, 106.2, 119.9, 122.6, 123.1, 124.0, 124.2, 124.7, 128.2, 129.6, 130.0, 130.6, 131.2, 133.2, 133.6, 134.0, 139.8, 139.9, 167.0; HRMS (ESI-TOF) *m/z*: $[M + H]^+$ Calcd for $[C_{21}H_{14}BrN_2O]$: 389.0311, Found 389.0307.

2-(4-Ethylphenyl)-4-phenylpyrrolo[2,3,4-*kl*]acridin-1(2*H*)-one (7c): Orange solid (88%), mp. 240-242 ºC (EtOAc); IR (KBr, υ cm^{-1}): 2942, 1705, 1599, 1205, 1044, 777; ^{1}H NMR (300 MHz, $CDCl_3$): δ (ppm) 1.33 (t, 3H, J=7.6 Hz), 2.77 (q, 2H, J=7.5 Hz), 7.23 (s, 1H), 7.43 (d, 3H, J=8.0 Hz), 7.50 (t, 2H, J=7.2 Hz), 7.56 (d, 2H, J=8.0 Hz), 7.71 (d, 2H, J=7.6 Hz), 7.79 (t, 1H, J=7.6 Hz), 7.92 (t, 1H, J=7.6 Hz), 8.05 (s, 1H), 8.43 (d, 1H, J=8.8 Hz), 8.90 (d, 1H, J=8.4 Hz); ^{13}C NMR (100 MHz, $CDCl_3$) 15.6, 28.8, 106.9, 119.5, 120.1, 123.1, 124.4, 126.1, 127.6, 127.8, 128.6, 129.2, 129.2, 130.8, 130.9, 132.4, 140.8, 141.1, 144.2, 146.7, 146.8, 152.5, 167.6; HRMS (ESI-TOF) *m/z*: $[M + H]^+$ Calcd for $[C_{28}H_{21}N_2O]$: 401.1643, Found 401.1645.

Copies of ^{1}H and ^{13}C NMR spectra of compound 4a–p and 7a–c:

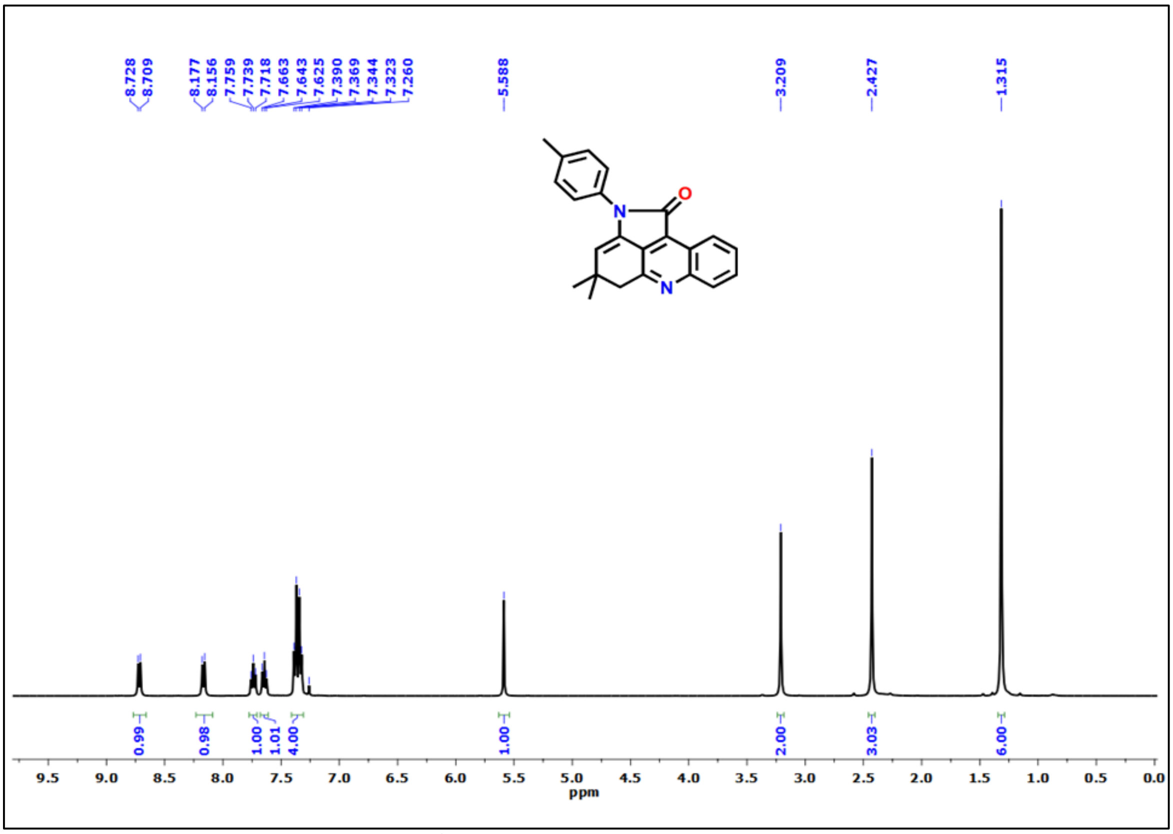

Figure S5: 400 MHz ^{1}H NMR spectrum of compound **4a** in $CDCl_3$

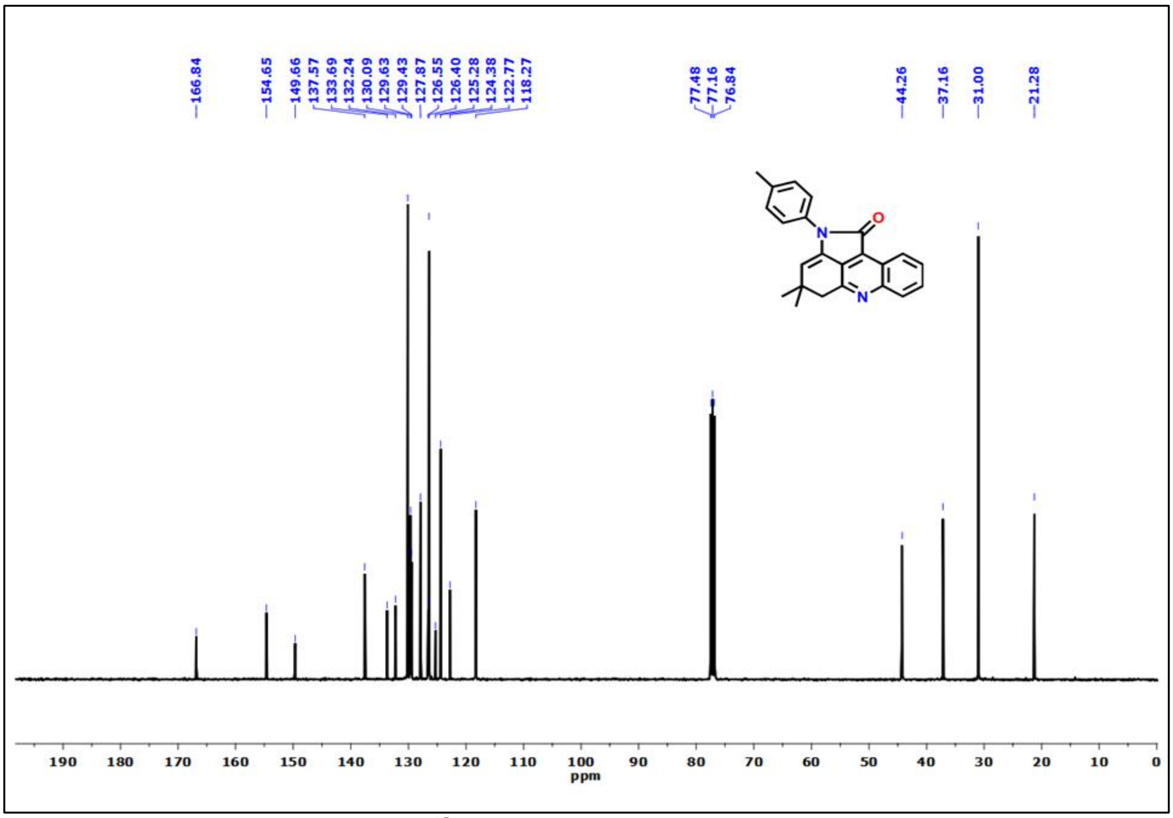

Figure S6: 100 MHz ^{13}C NMR spectrum of compound **4a** in $CDCl_3$

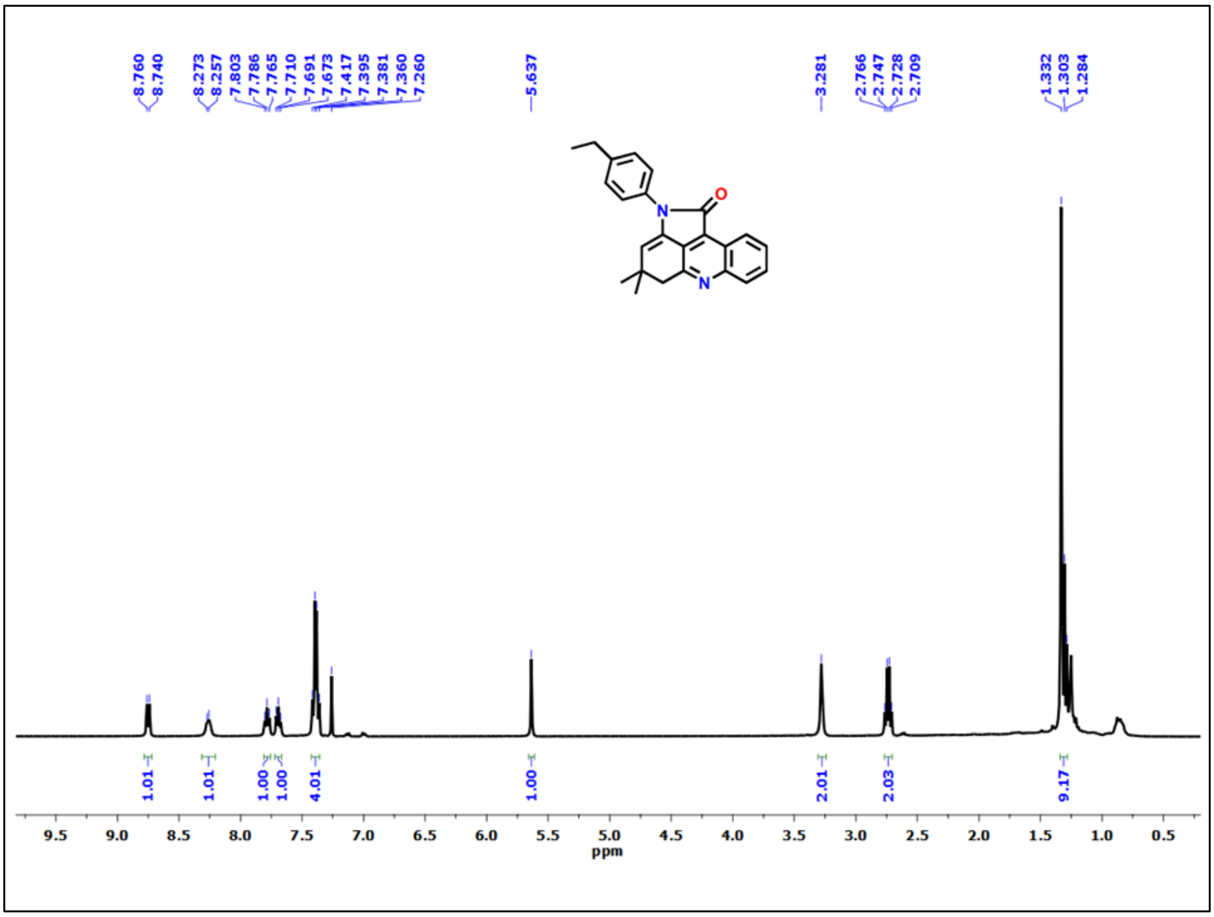

Figure S7: 400 MHz ^{1}H NMR spectrum of compound **4b** in $CDCl_3$

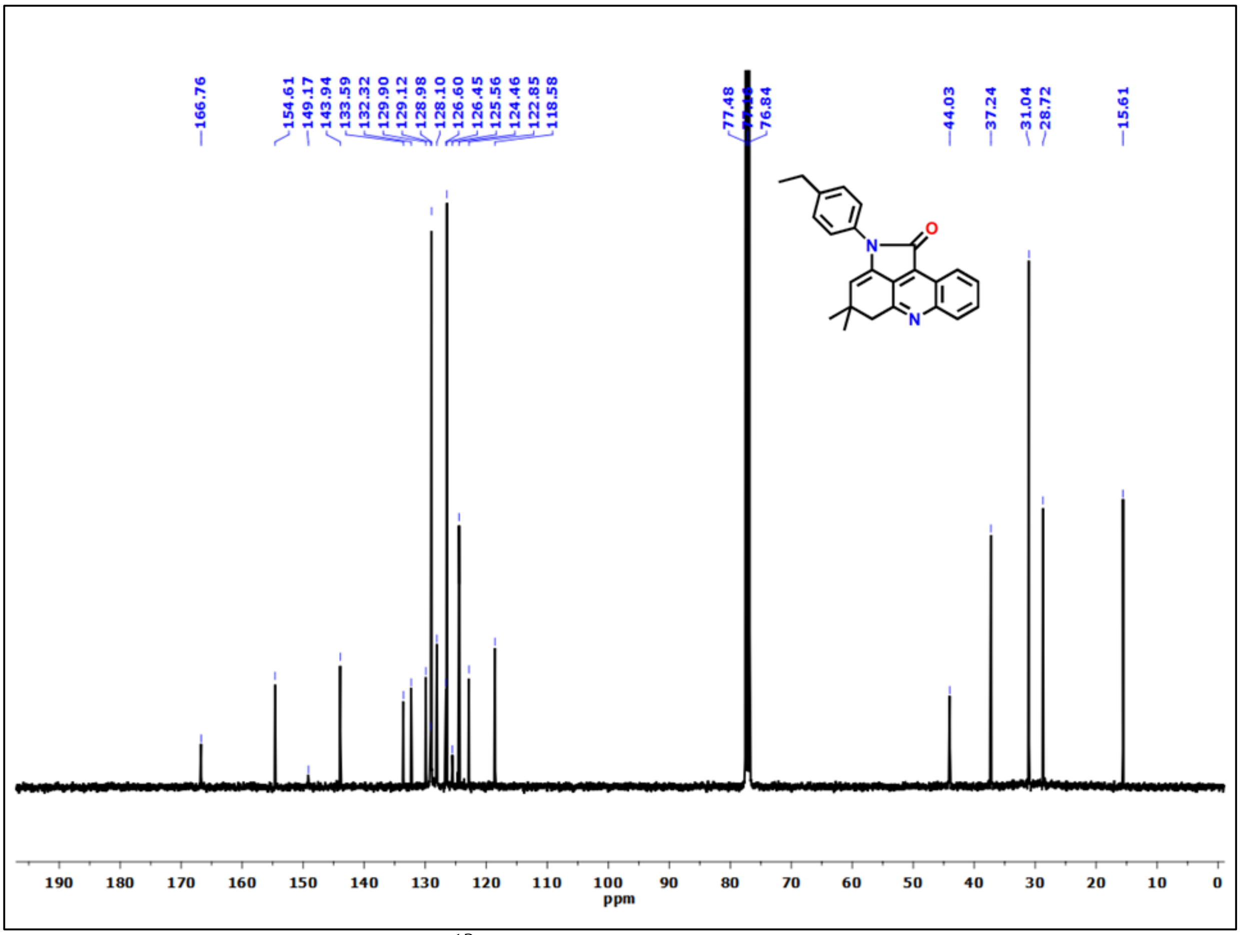

Figure S8: 100 MHz ^{13}C NMR spectrum of compound **4b** in $CDCl_3$

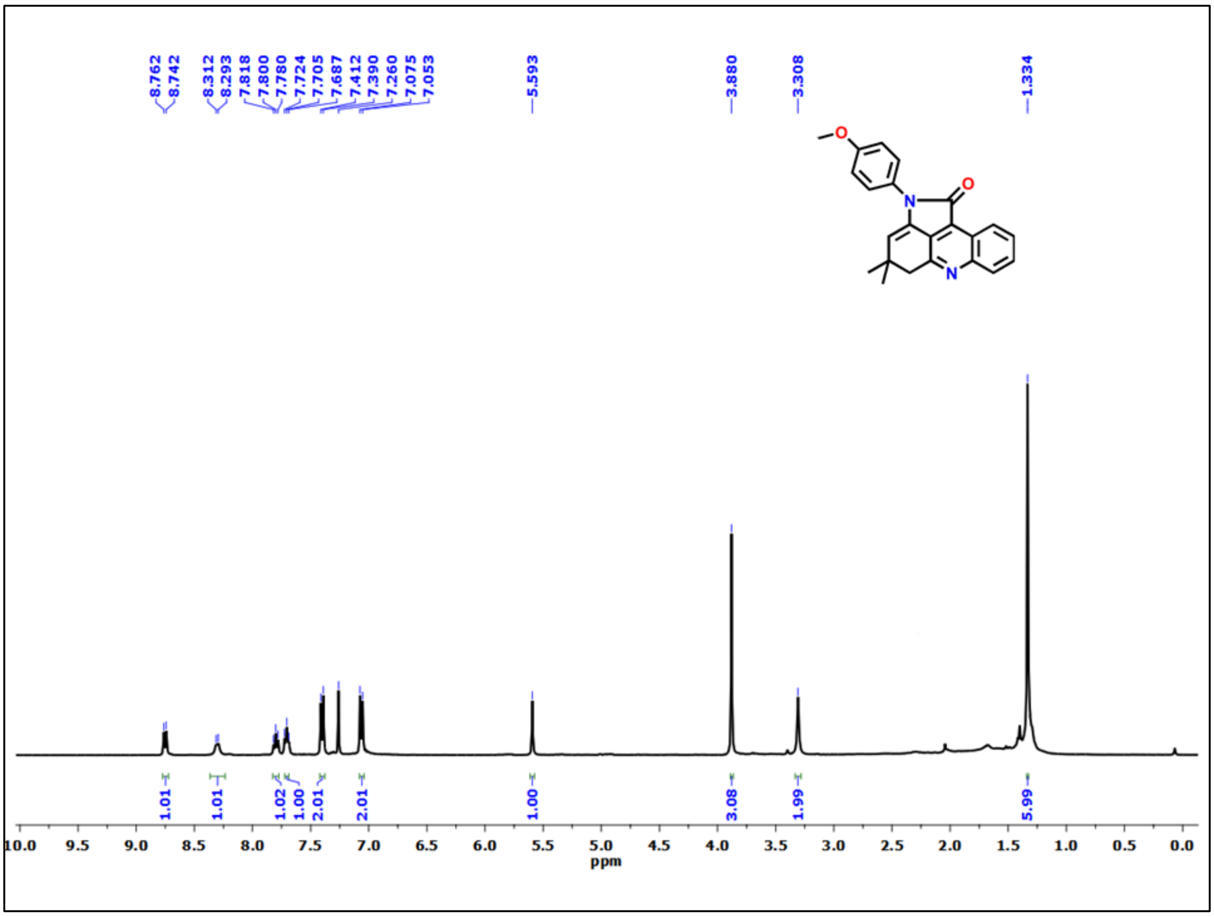

Figure S9: 400 MHz ^{1}H NMR spectrum of compound **4c** in $CDCl_3$

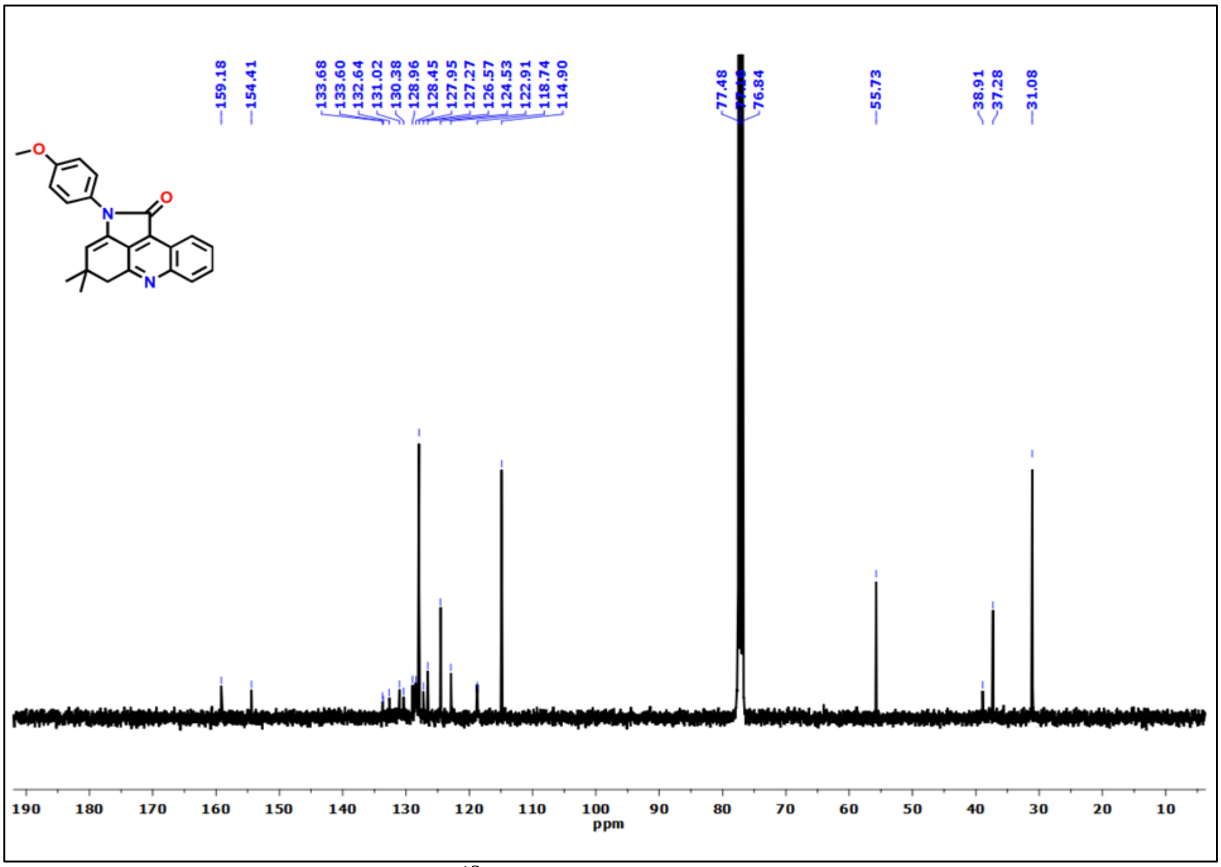

Figure S10: 100 MHz ^{13}C NMR spectrum of compound **4c** in $CDCl_3$

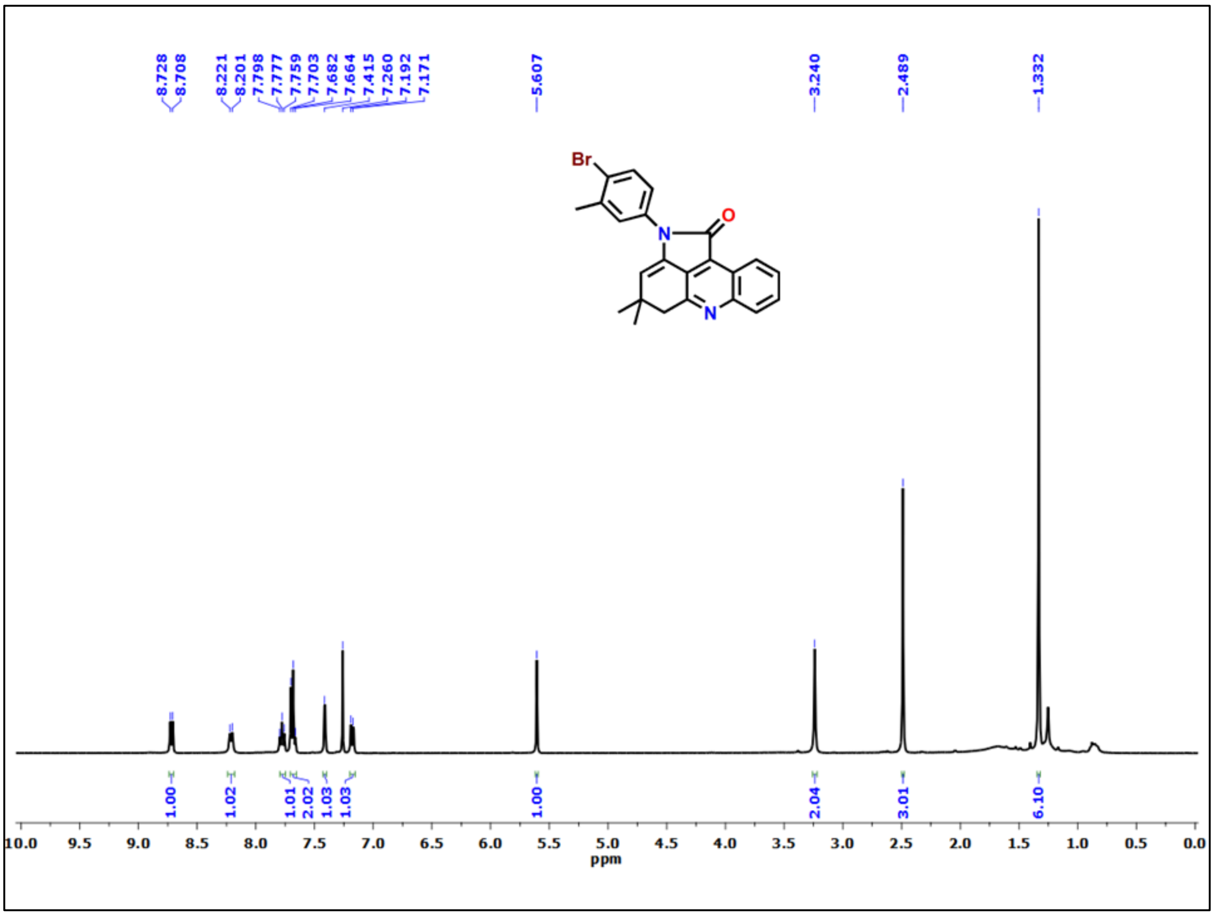

Figure S11: 400 MHz ^{1}H NMR spectrum of compound **4d** in $CDCl_3$

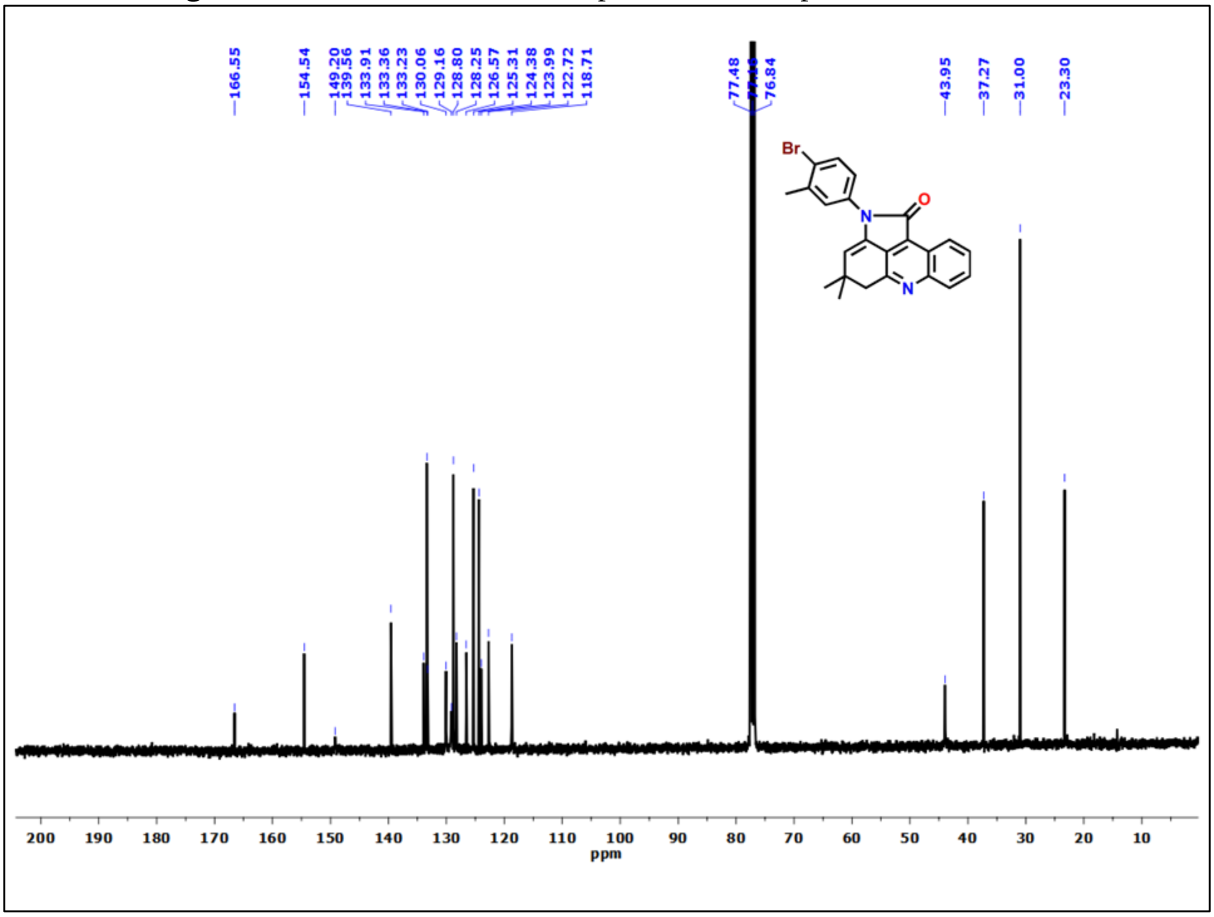

Figure S12: 100 MHz ^{13}C NMR spectrum of compound **4d** in $CDCl_3$

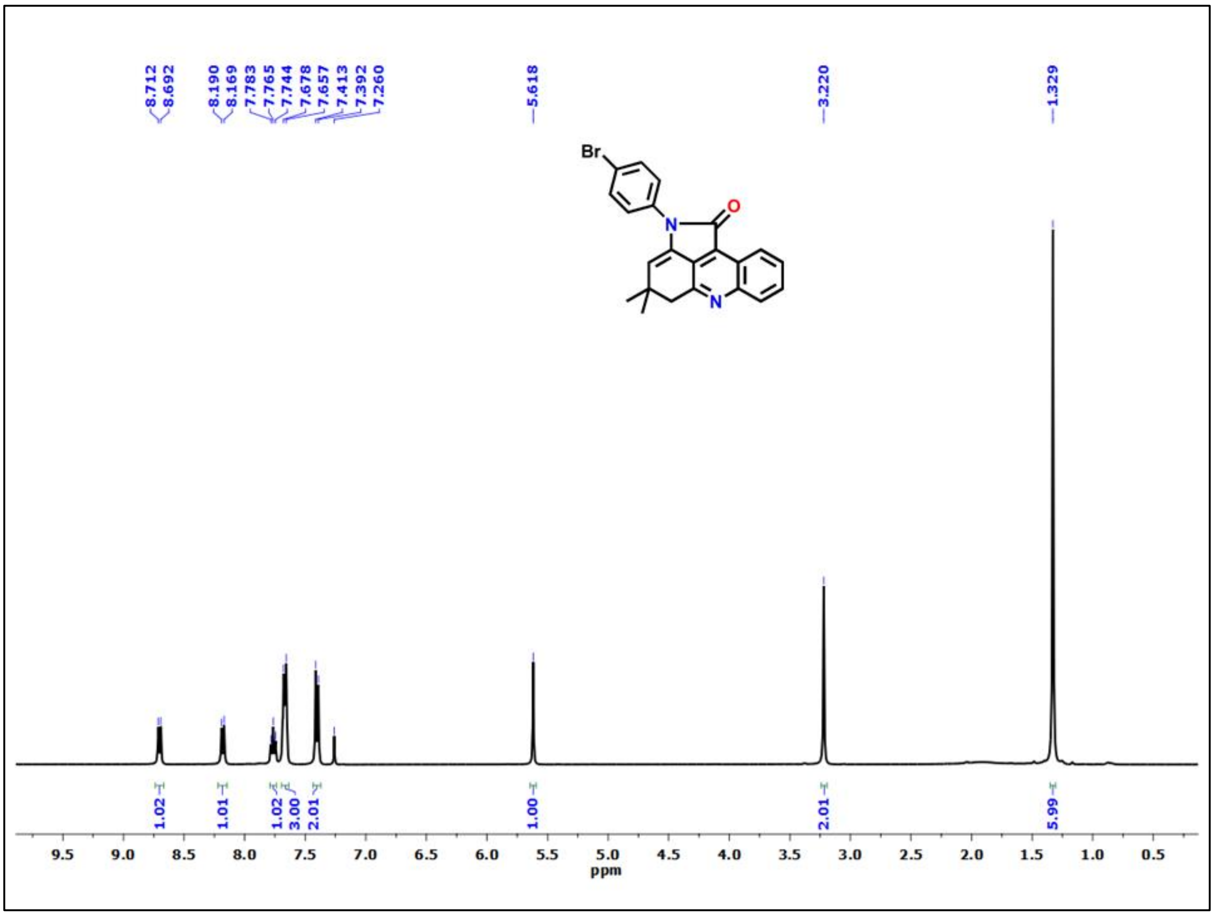

Figure S13: 400 MHz ^{1}H NMR spectrum of compound **4e** in $CDCl_3$

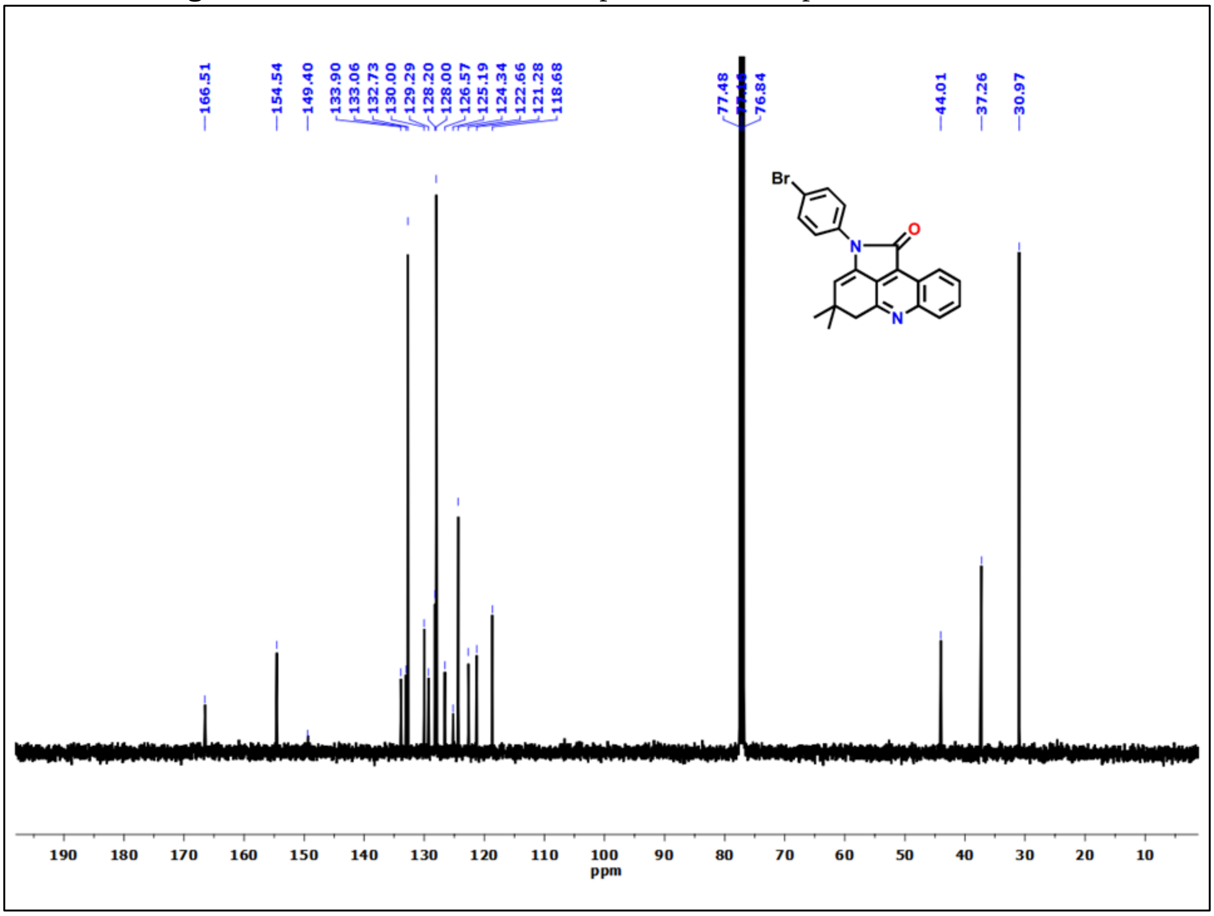

Figure S14: 100 MHz ^{13}C NMR spectrum of compound **4e** in $CDCl_3$

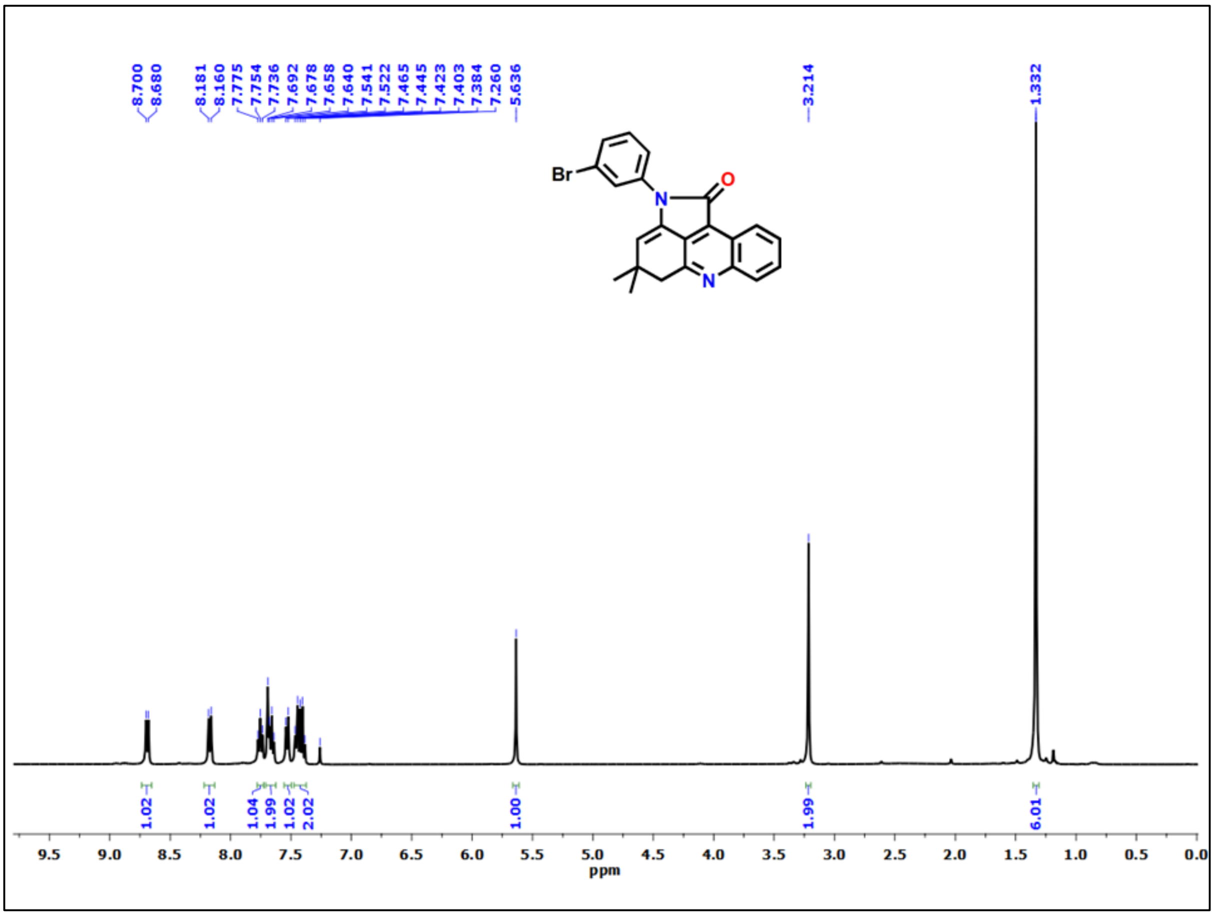

Figure S15: 400 MHz ^{1}H NMR spectrum of compound **4f** in $CDCl_3$

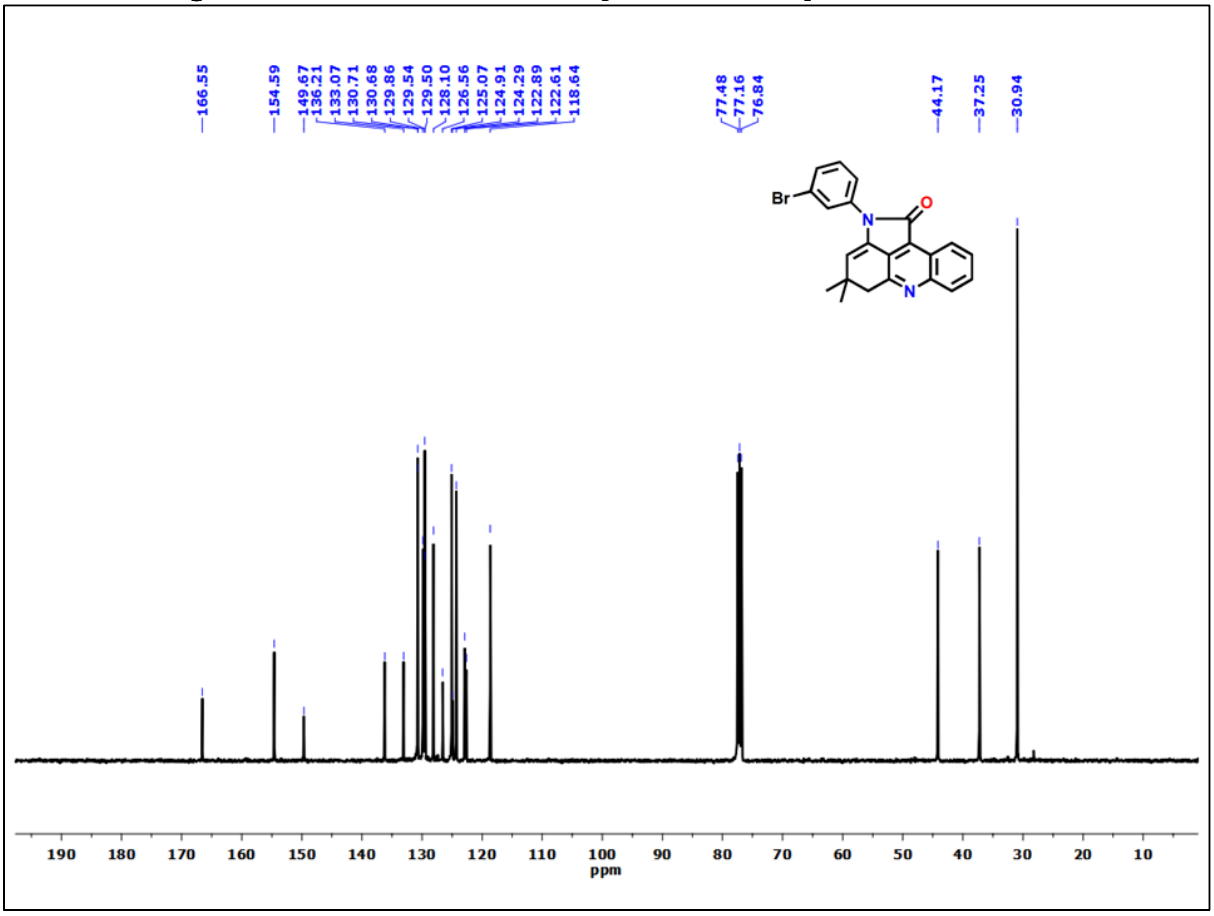

Figure S16: 100 MHz ^{13}C NMR spectrum of compound **4f** in $CDCl_3$

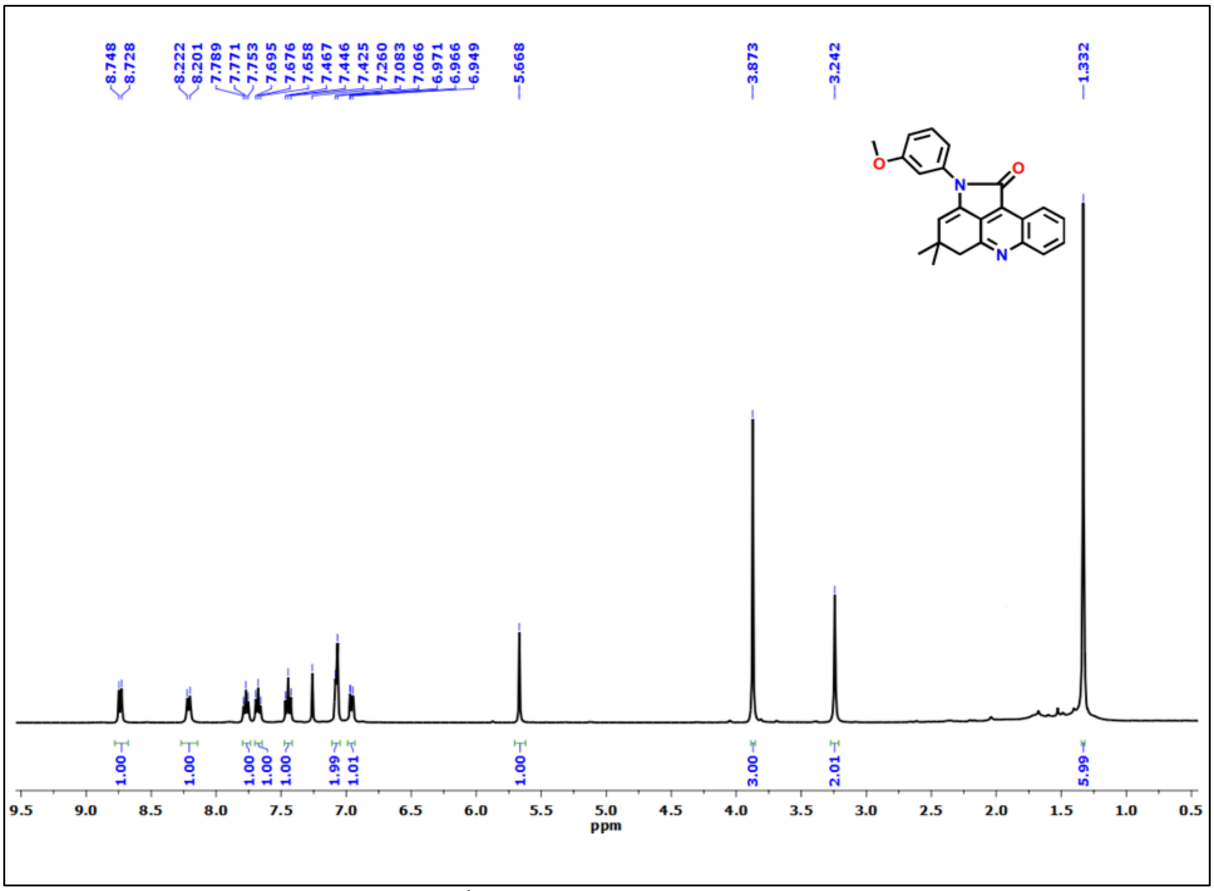

Figure S17: 400 MHz ^{1}H NMR spectrum of compound **4g** in $CDCl_3$

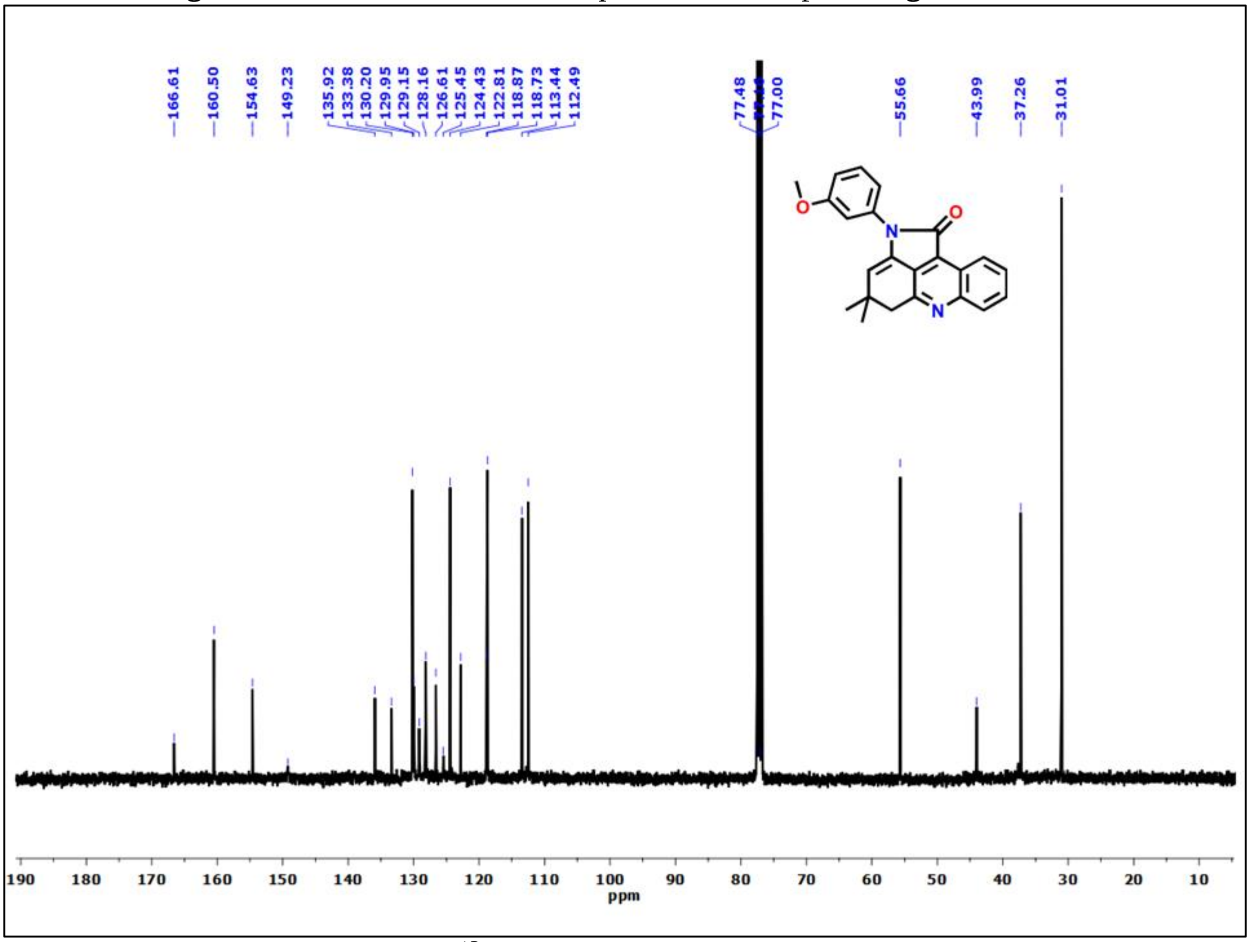

Figure S18: 100 MHz ^{13}C NMR spectrum of compound **4g** in $CDCl_3$

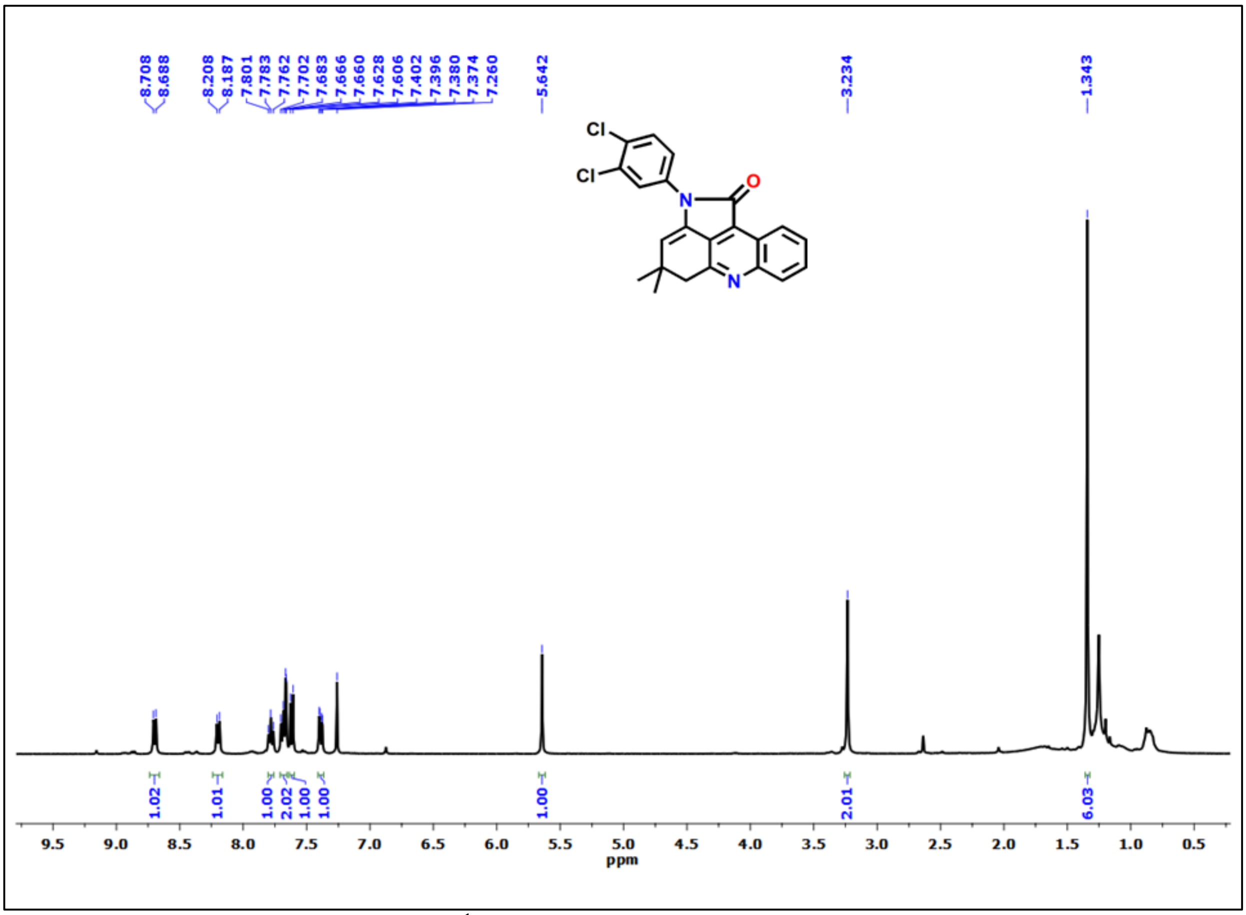

Figure S19: 400 MHz ^{1}H NMR spectrum of compound **4h** in $CDCl_3$

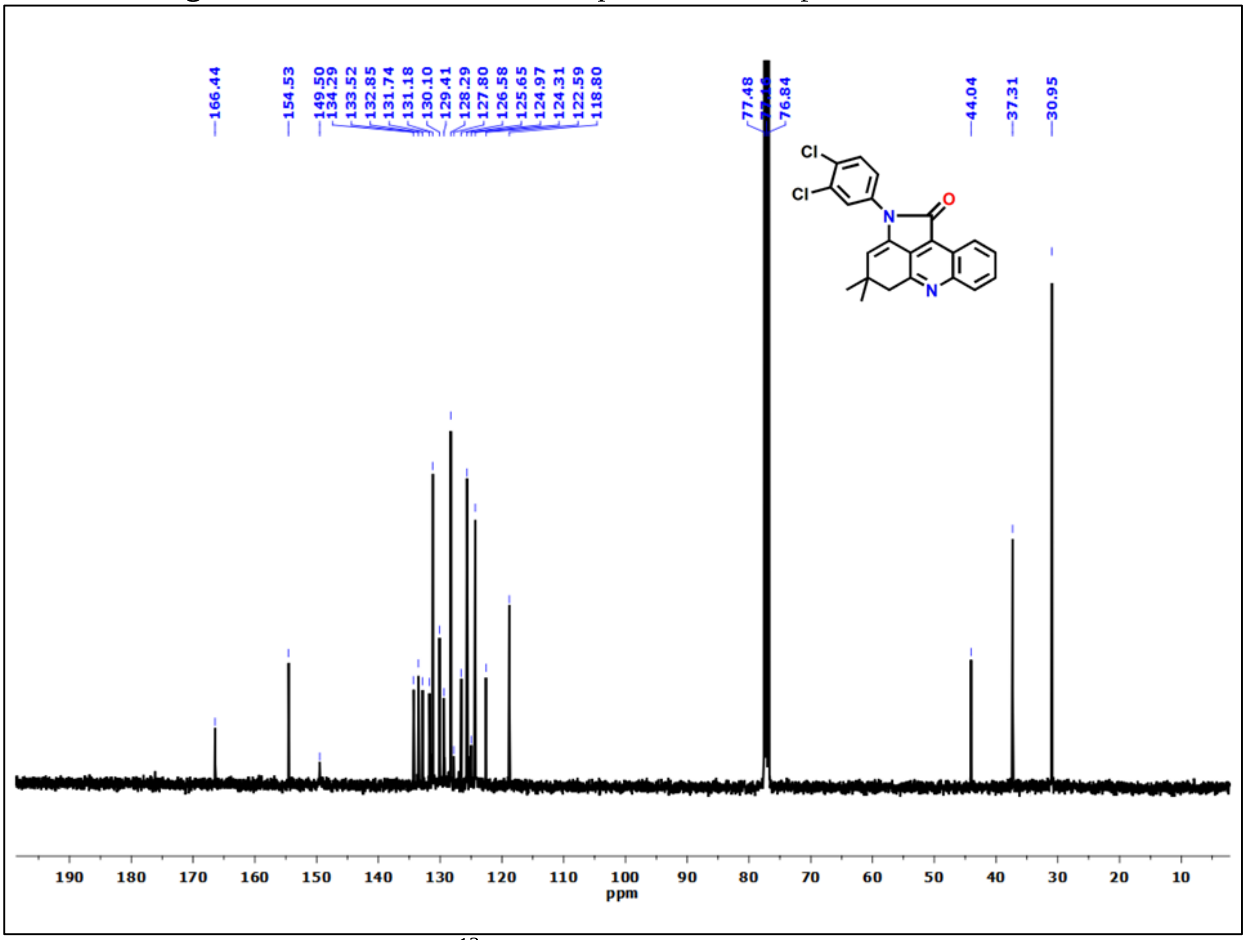

Figure S20: 100 MHz ^{13}C NMR spectrum of compound **4h** in $CDCl_3$

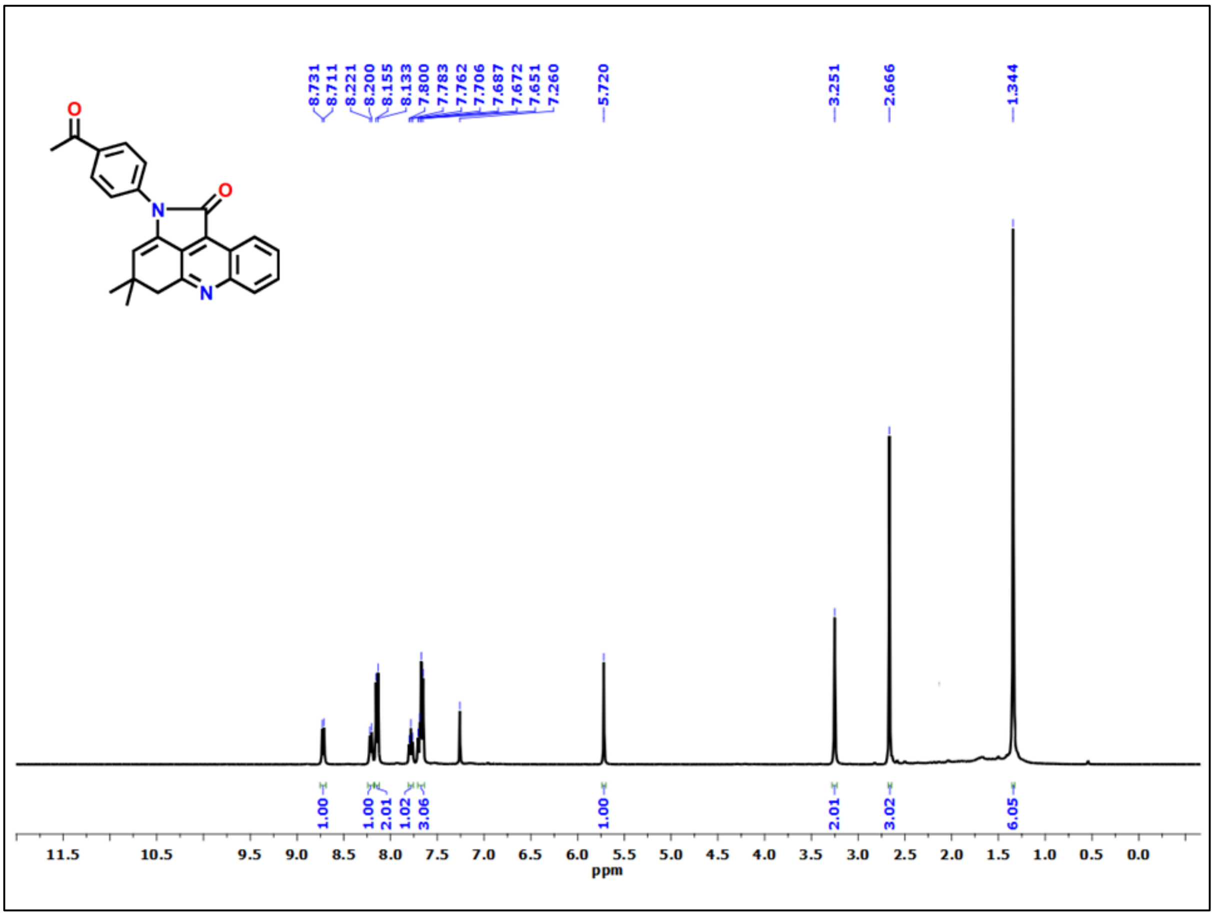

Figure S21: 400 MHz ^{1}H NMR spectrum of compound **4i** in $CDCl_3$

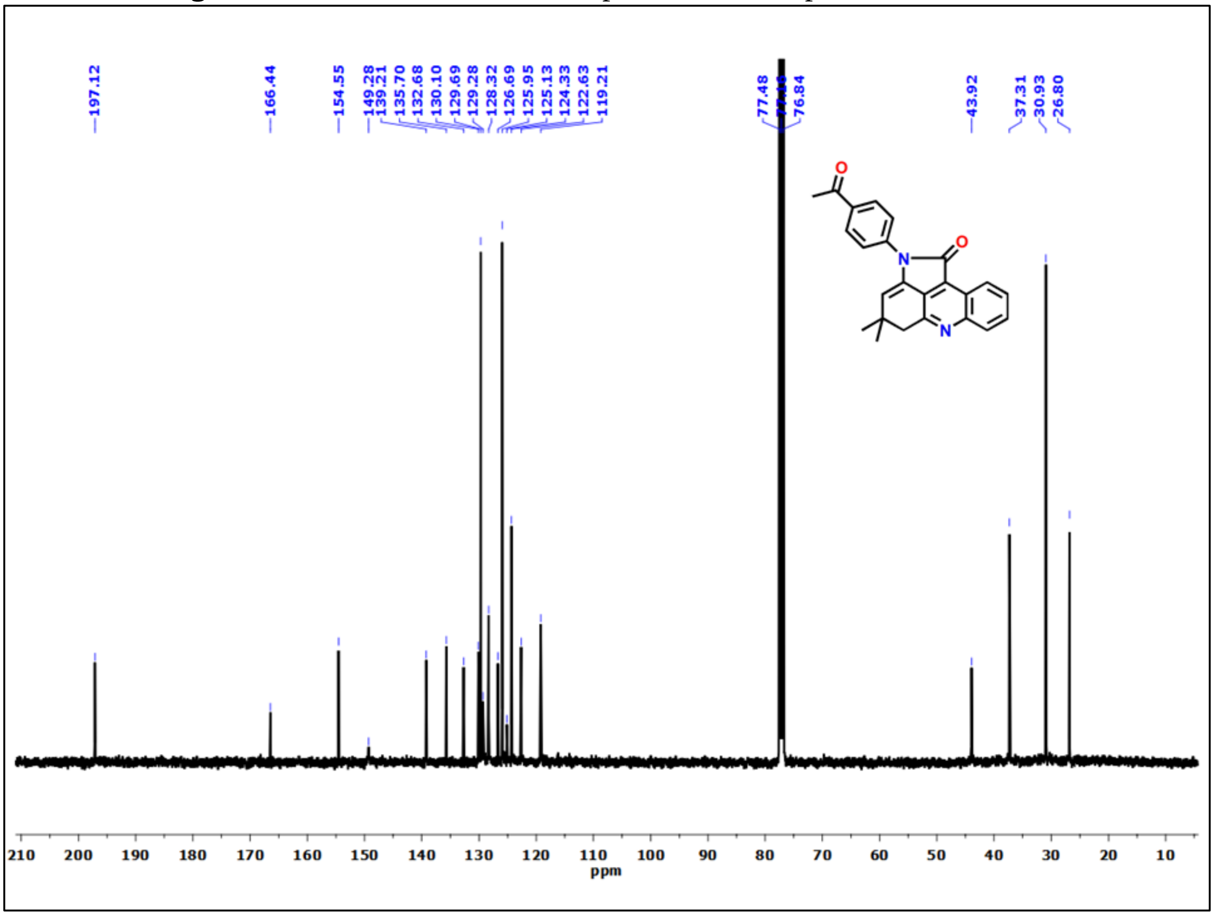

Figure S22: 100 MHz ^{13}C NMR spectrum of compound **4i** in $CDCl_3$

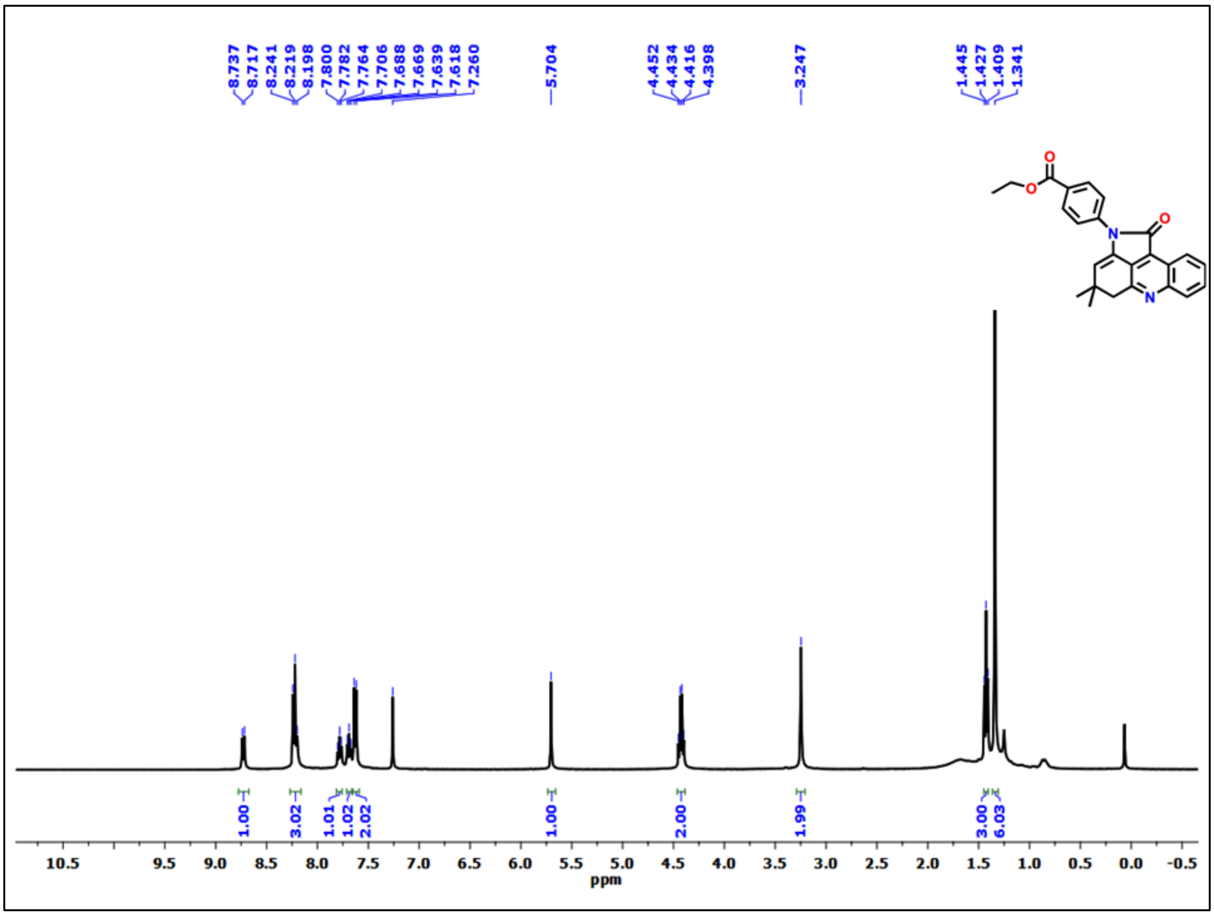

Figure S23: 400 MHz ^{1}H NMR spectrum of compound **4j** in $CDCl_3$

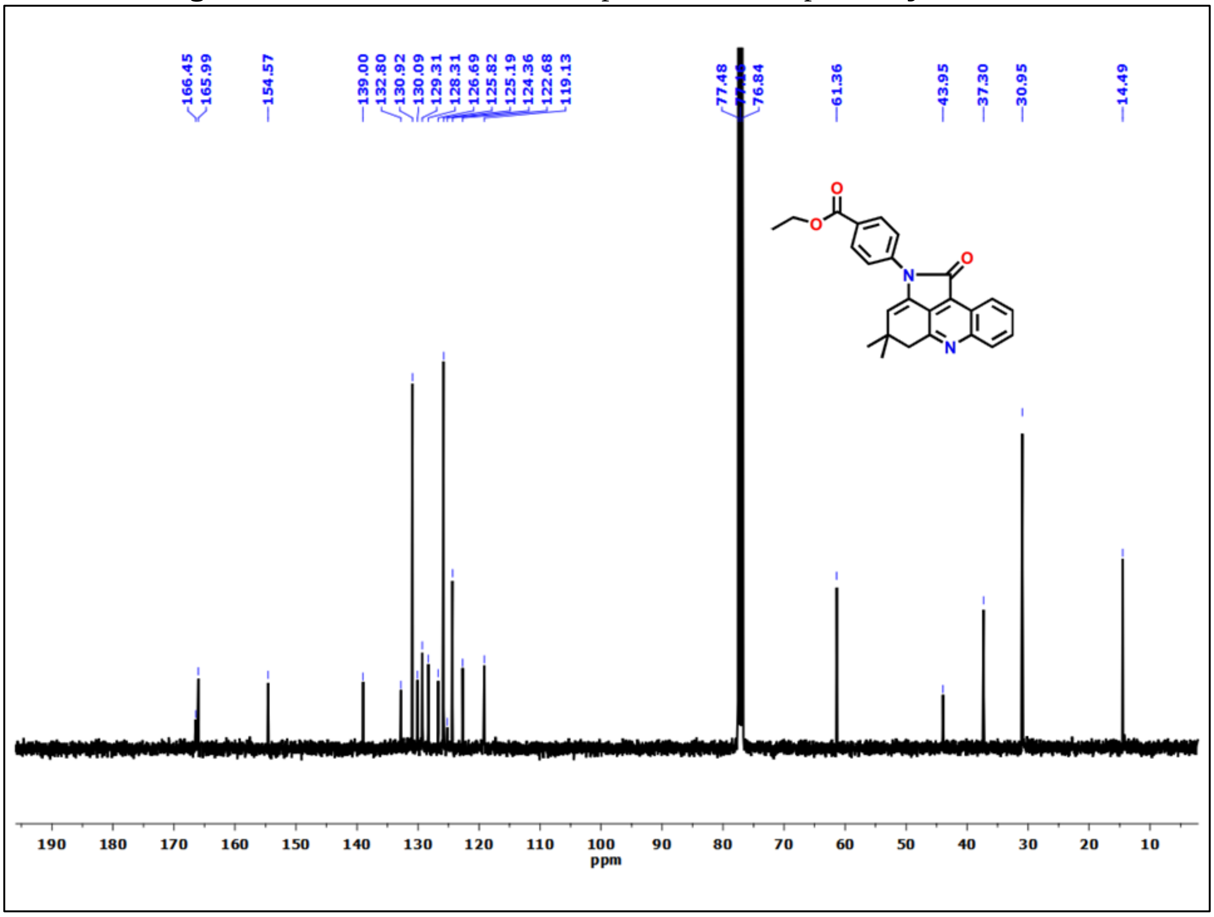

Figure S24: 100 MHz ^{13}C NMR spectrum of compound **4j** in $CDCl_3$

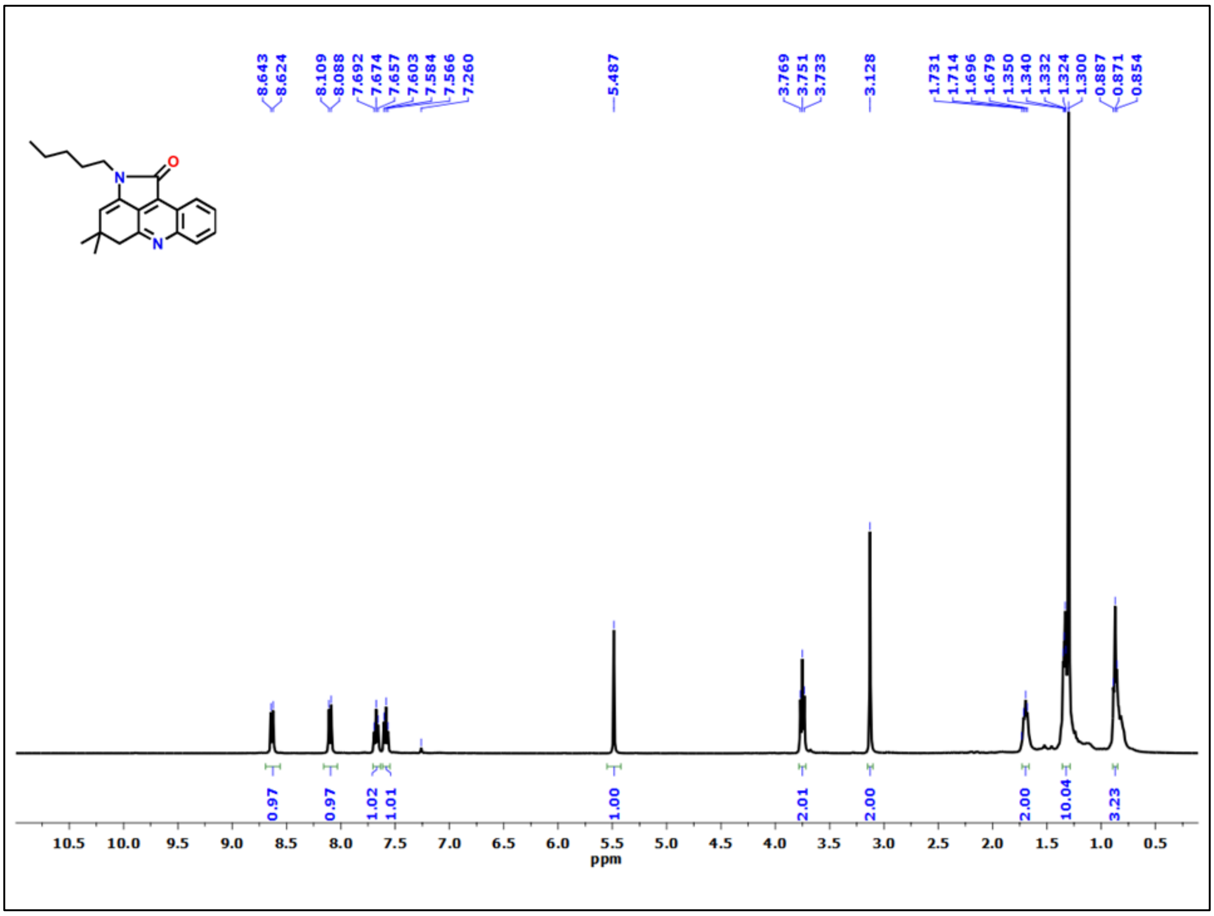

Figure S25: 400 MHz ^{1}H NMR spectrum of compound **4k** in $CDCl_3$

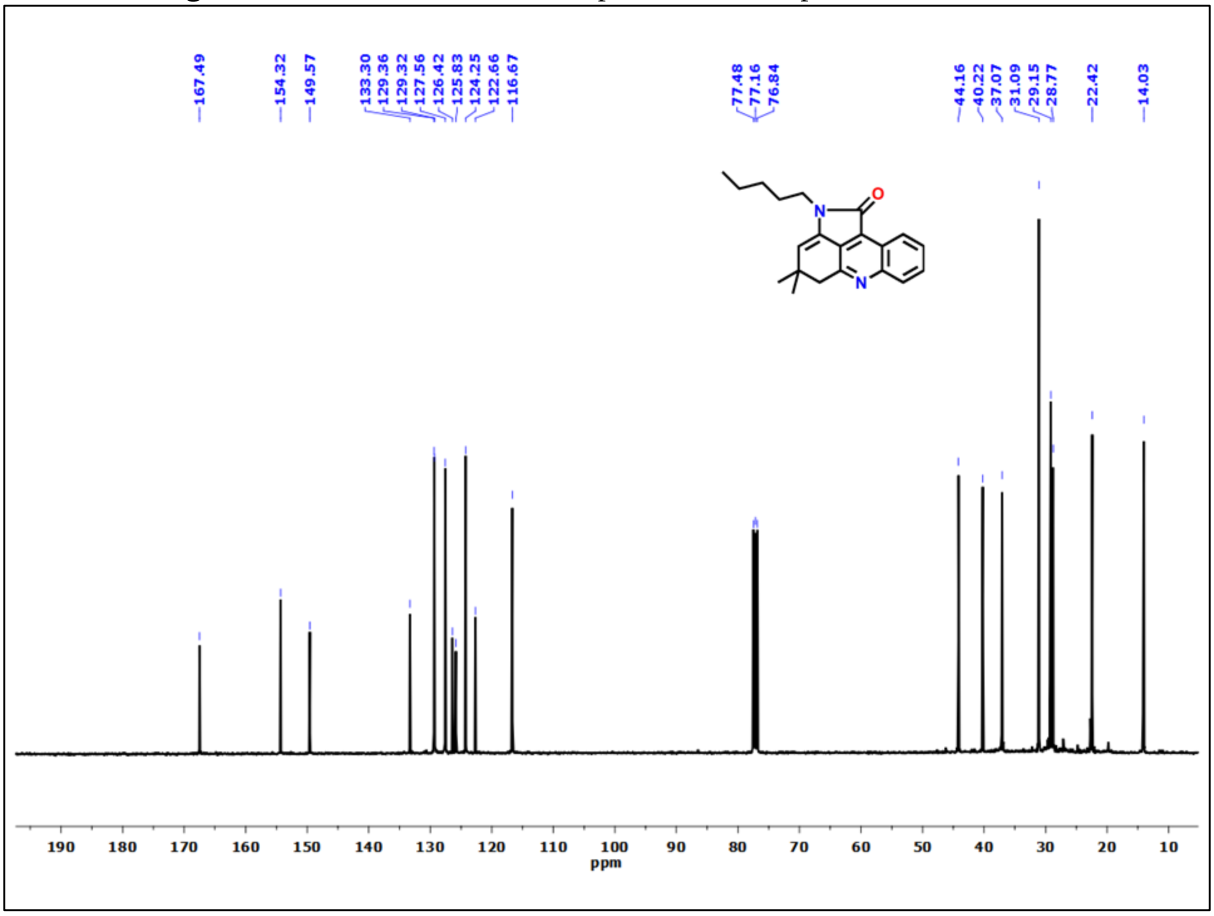

Figure S26: 100 MHz ^{13}C NMR spectrum of compound **4k** in $CDCl_3$

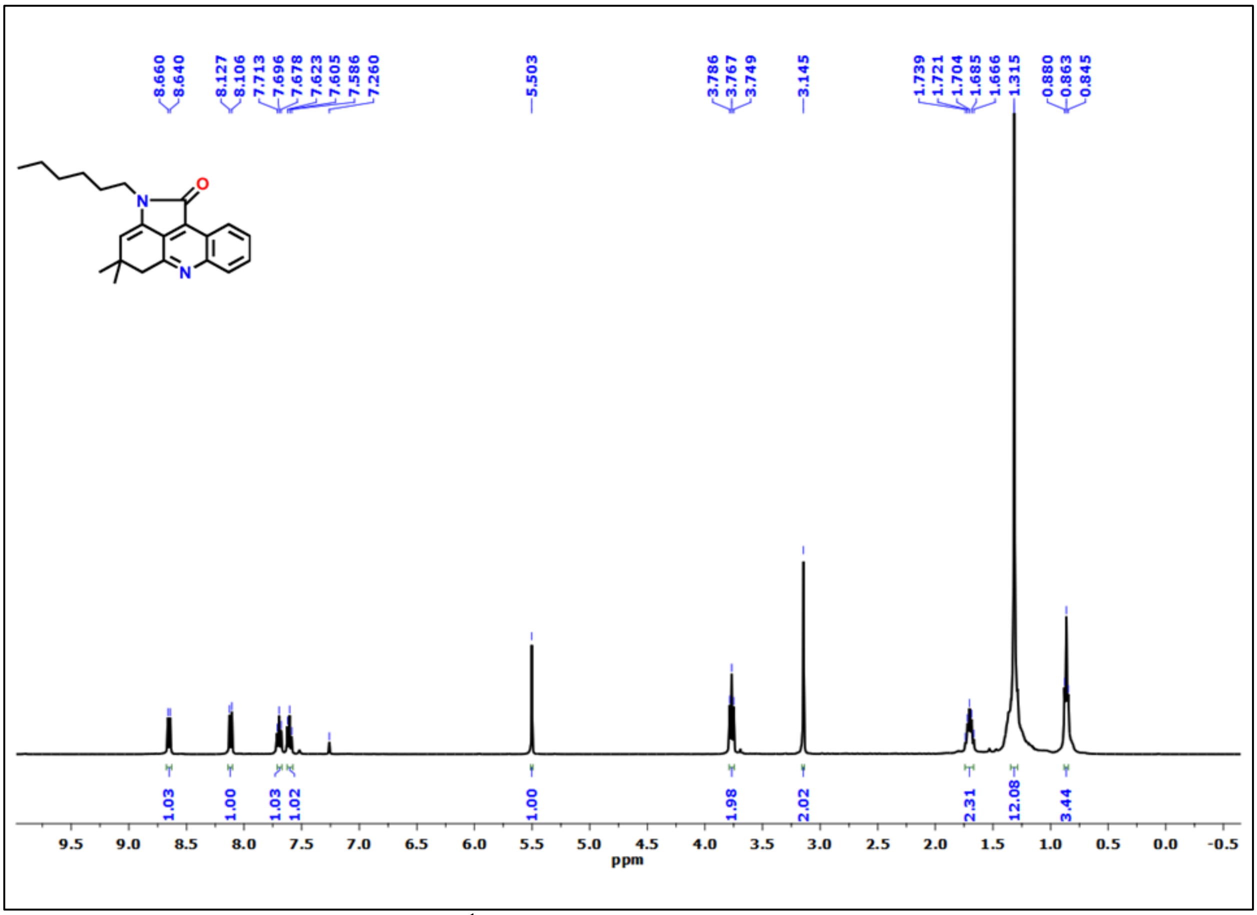

Figure S27: 400 MHz ^{1}H NMR spectrum of compound **4l** in $CDCl_3$

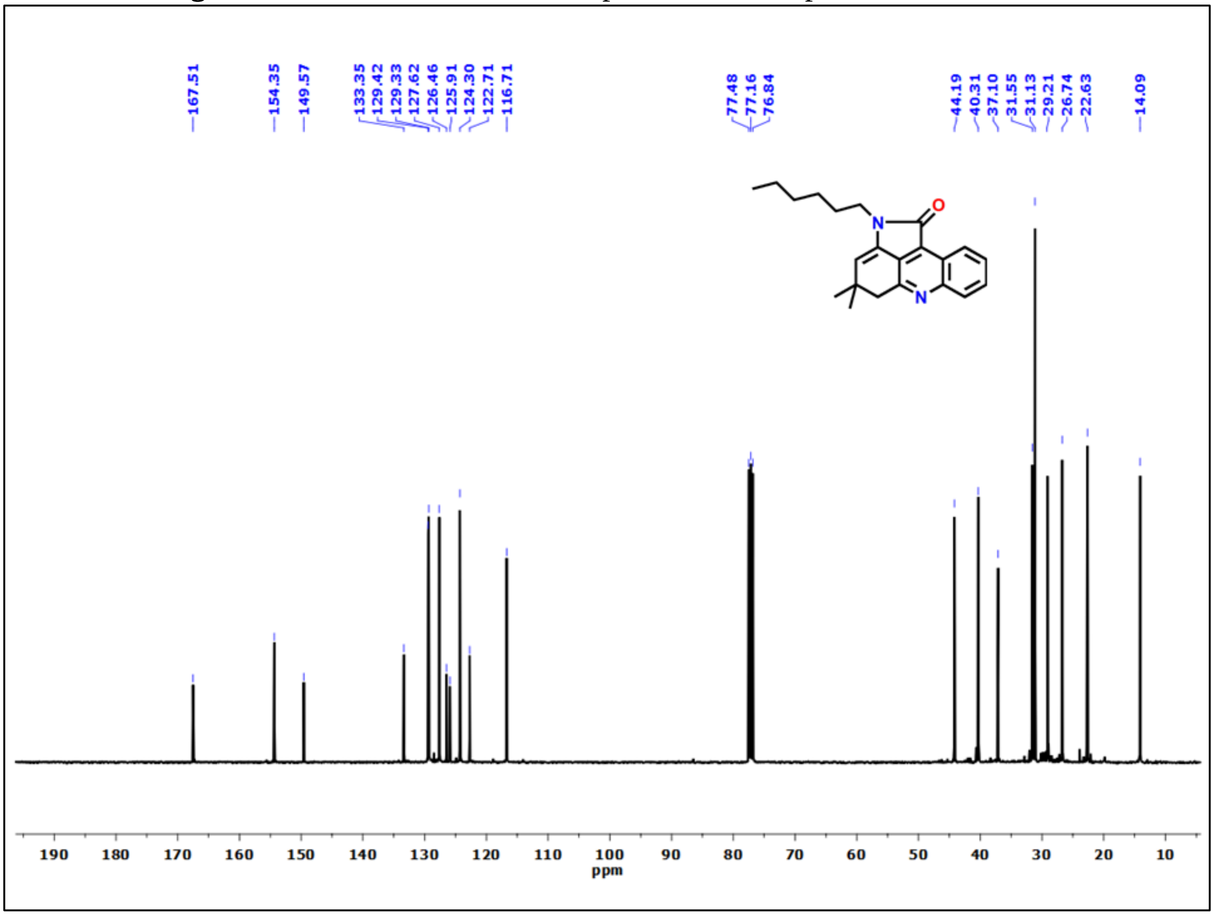

Figure S28: 100 MHz ^{13}C NMR spectrum of compound **4l** in $CDCl_3$

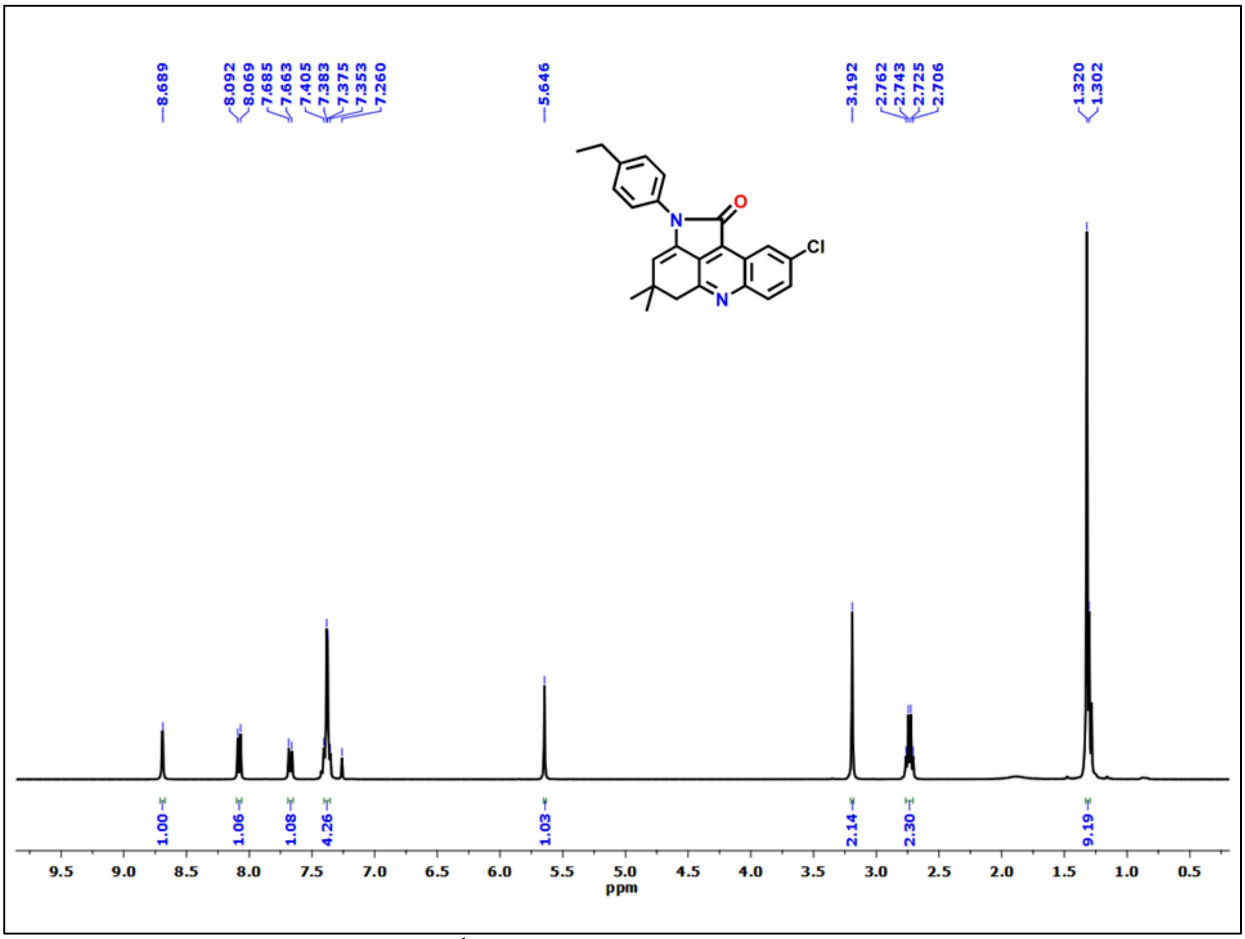

Figure S29: 400 MHz ^{1}H NMR spectrum of compound **4m** in $CDCl_3$

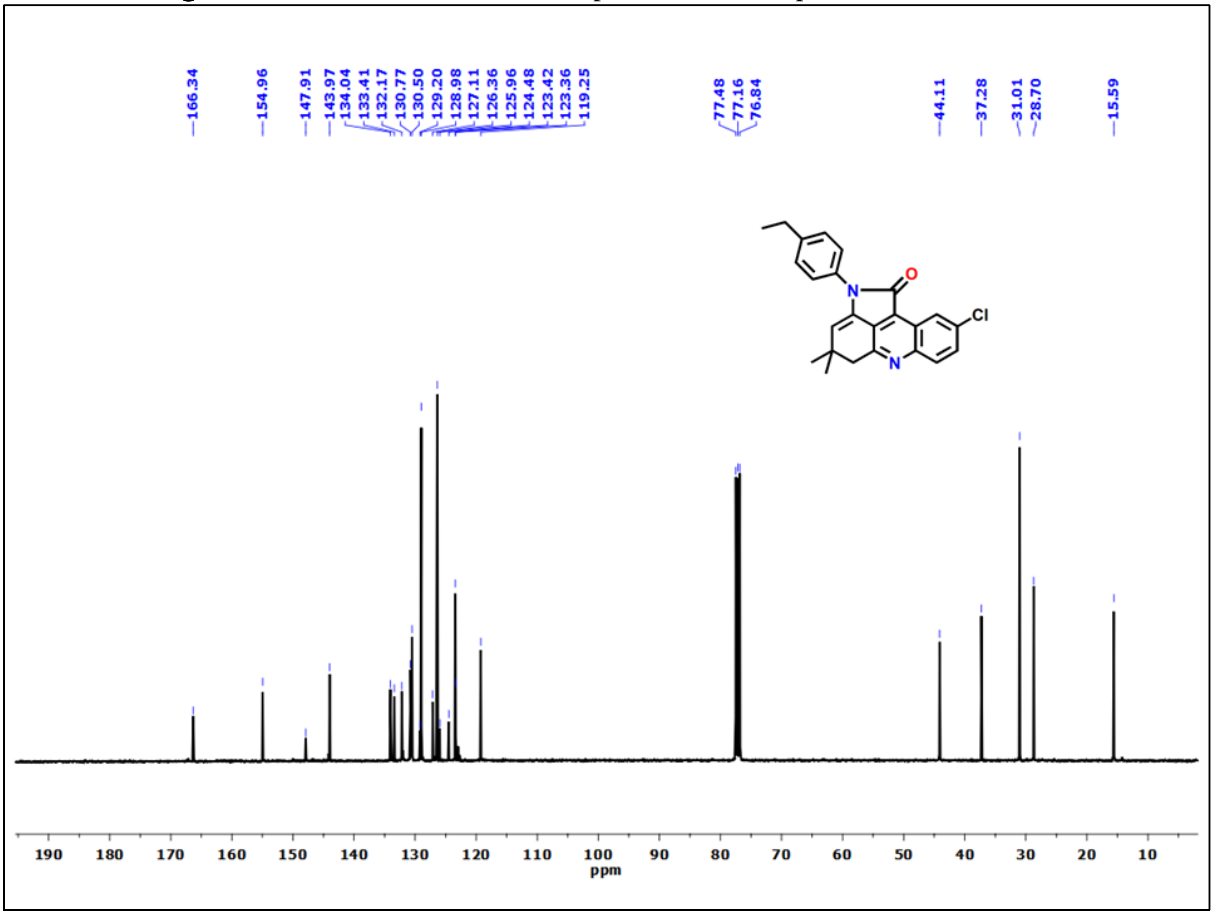

Figure S30: 100 MHz ^{13}C NMR spectrum of compound **4m** in $CDCl_3$

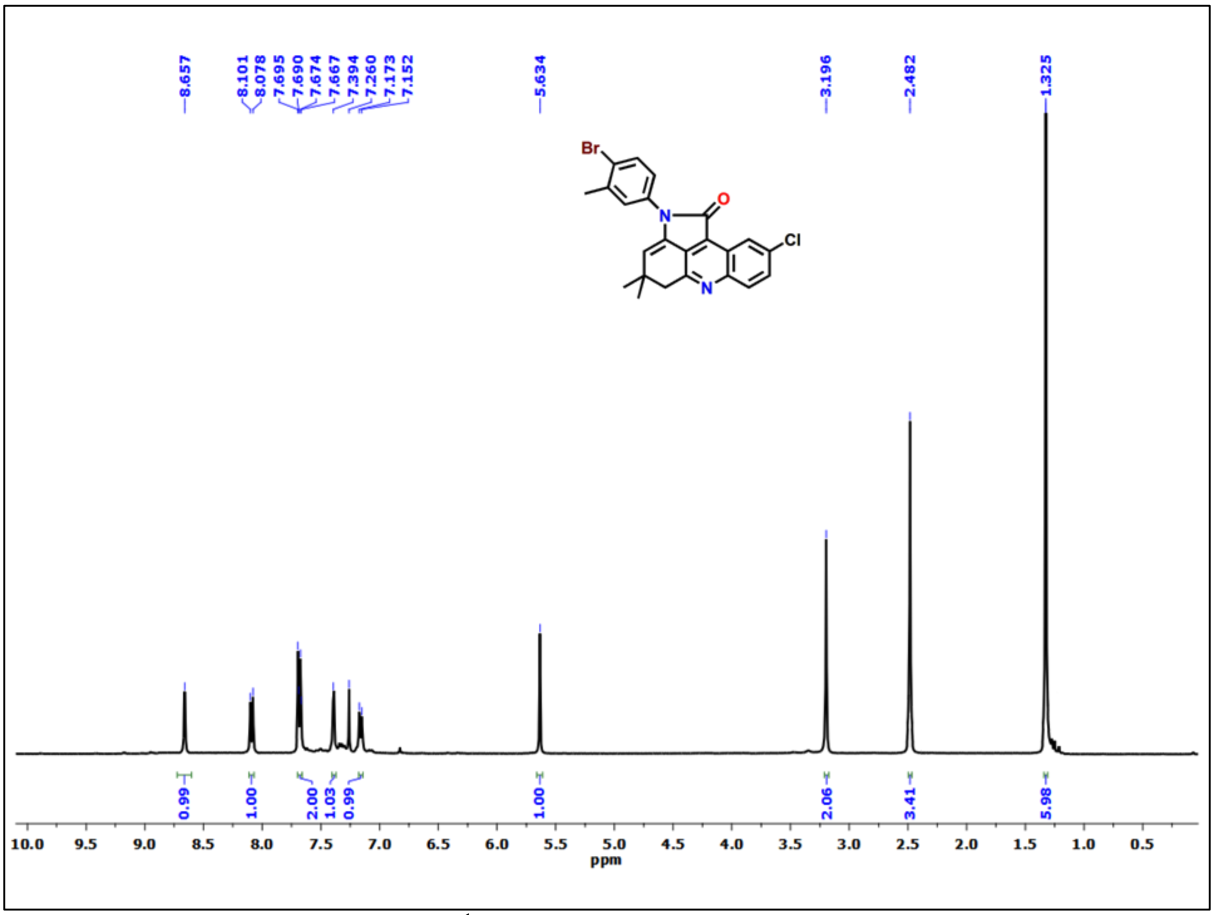

Figure S31: 400 MHz ^{1}H NMR spectrum of compound **4n** in $CDCl_3$

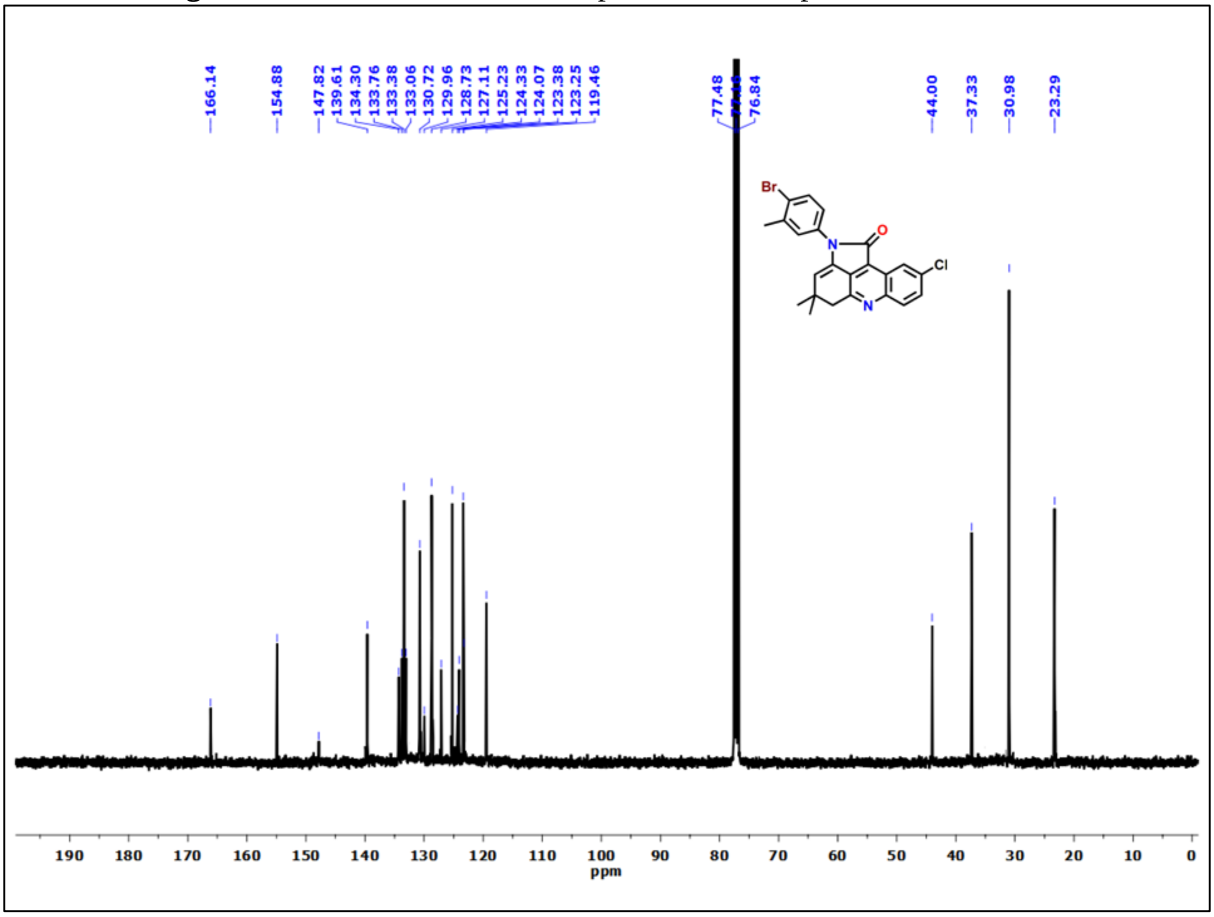

Figure S32: 100 MHz ^{13}C NMR spectrum of compound **4n** in $CDCl_3$

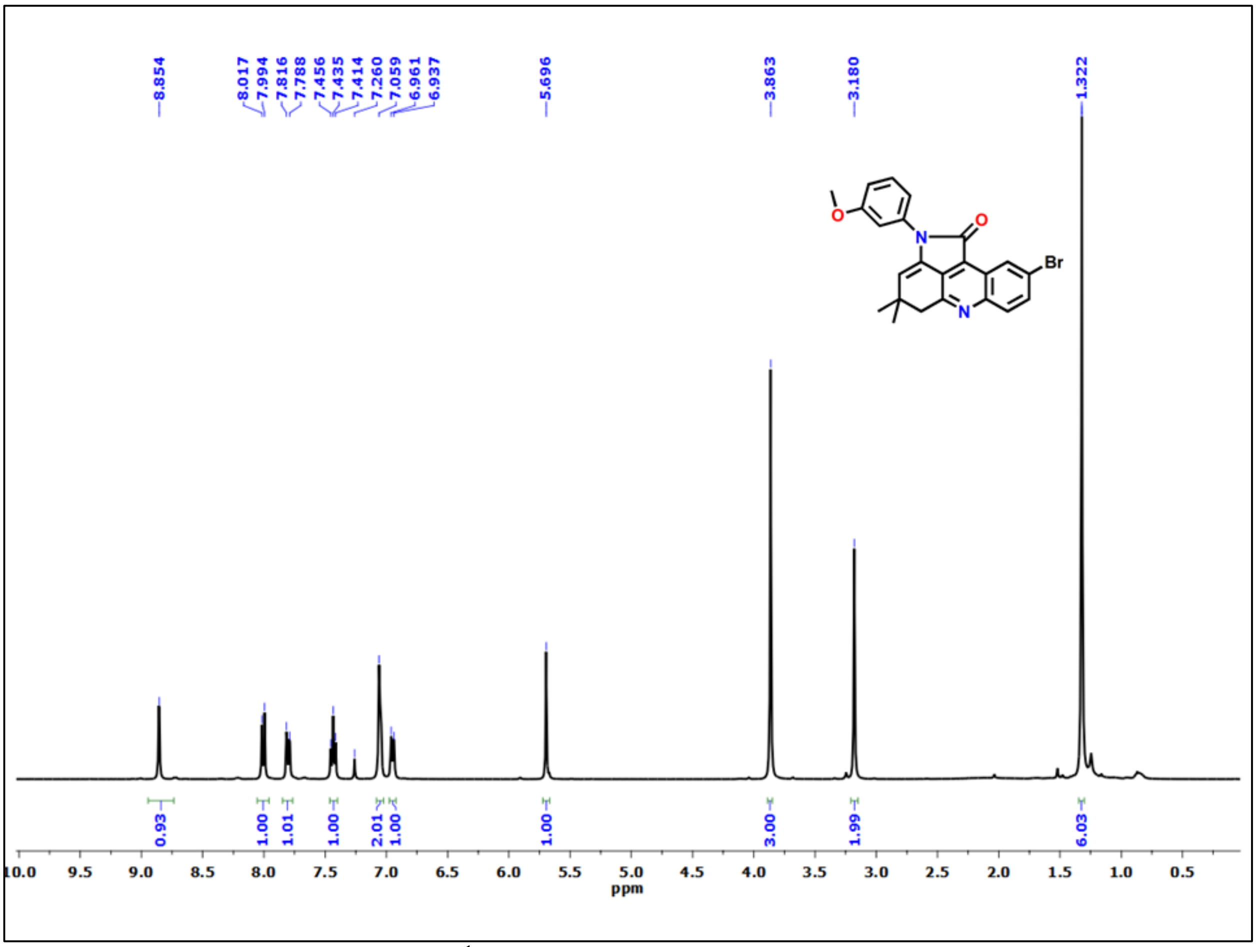

Figure S33: 400 MHz ^{1}H NMR spectrum of compound **4o** in $CDCl_3$

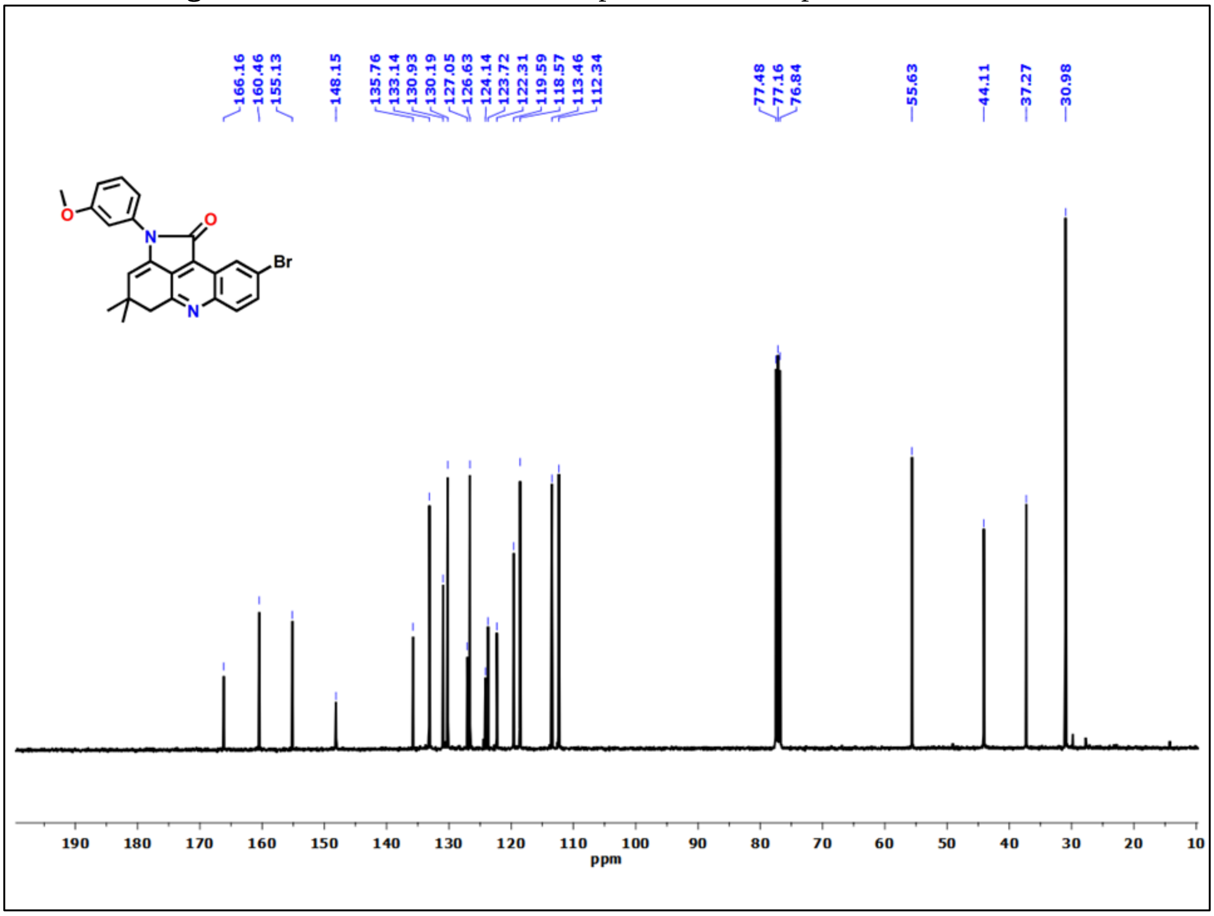

Figure S34: 100 MHz ^{13}C NMR spectrum of compound **4o** in $CDCl_3$

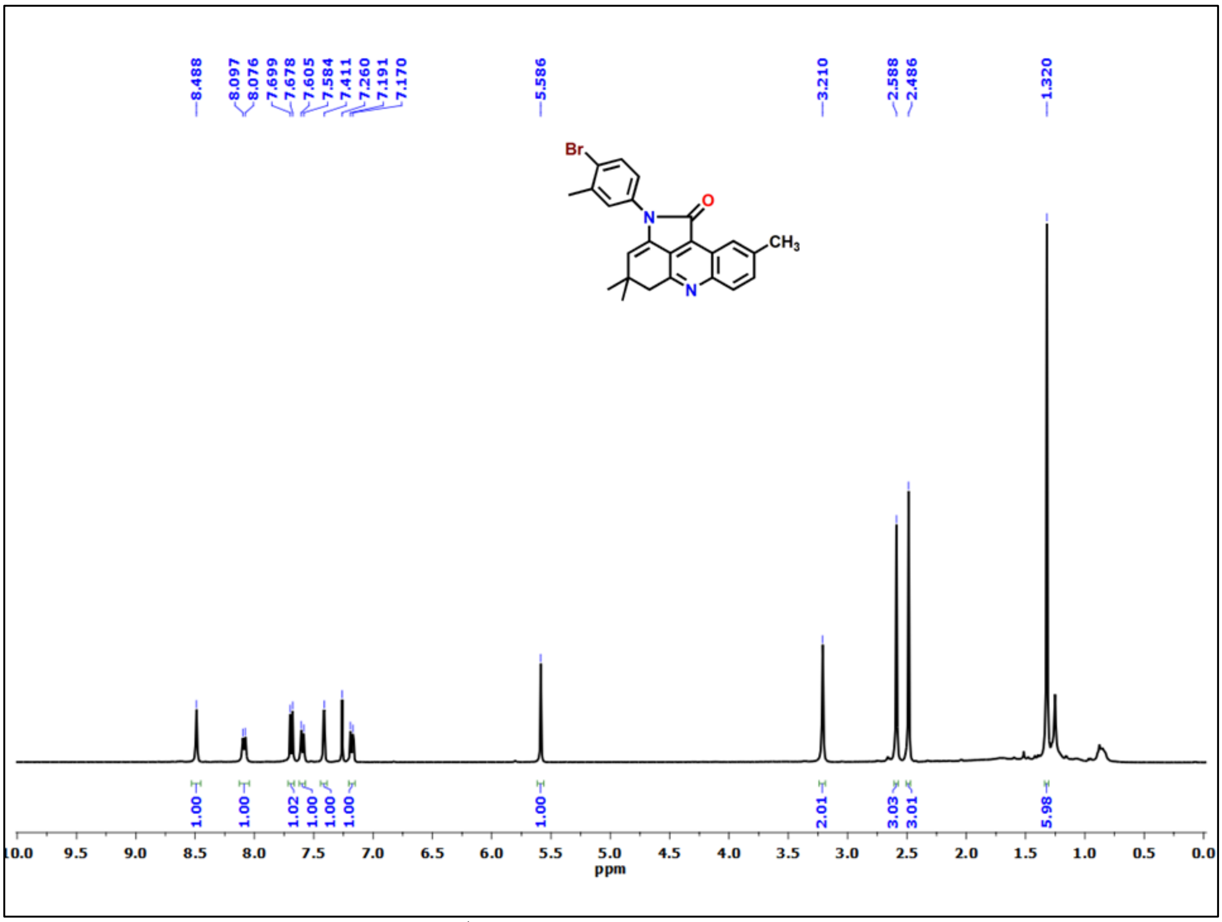

Figure S35: 400 MHz ^{1}H NMR spectrum of compound **4p** in $CDCl_3$

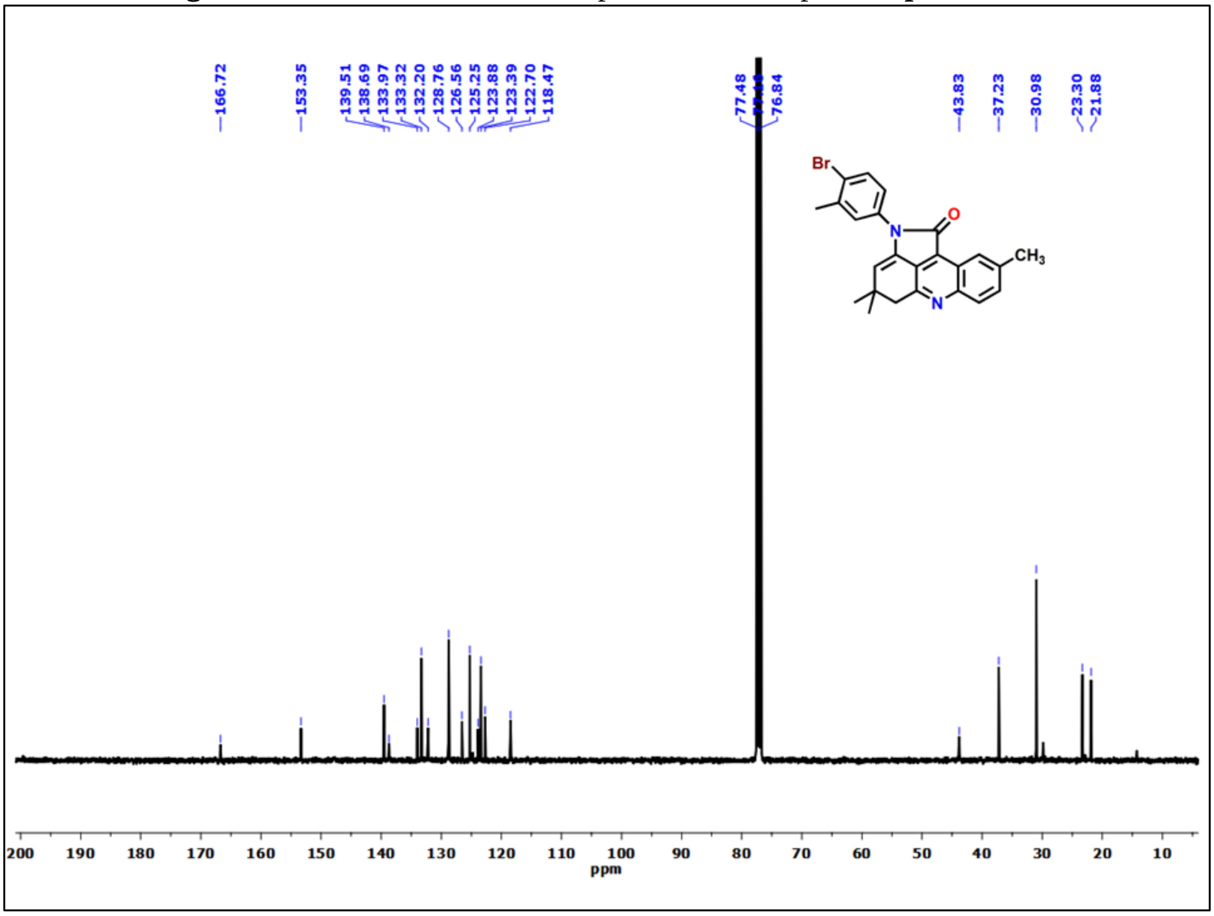

Figure S36: 100 MHz ^{13}C NMR spectrum of compound **4p** in $CDCl_3$

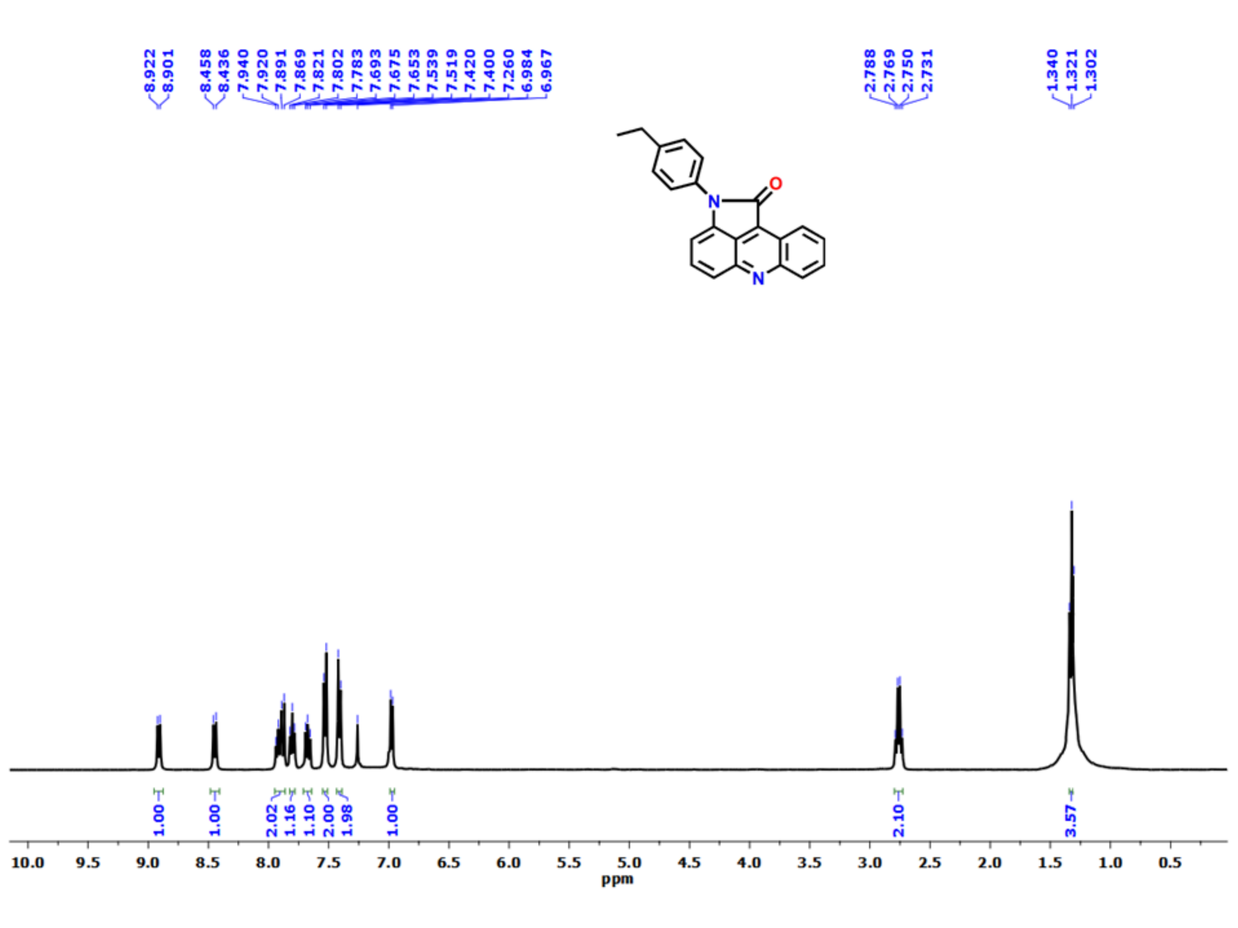

Figure S37: 400 MHz ^{1}H NMR spectrum of compound **7a** in $CDCl_3$

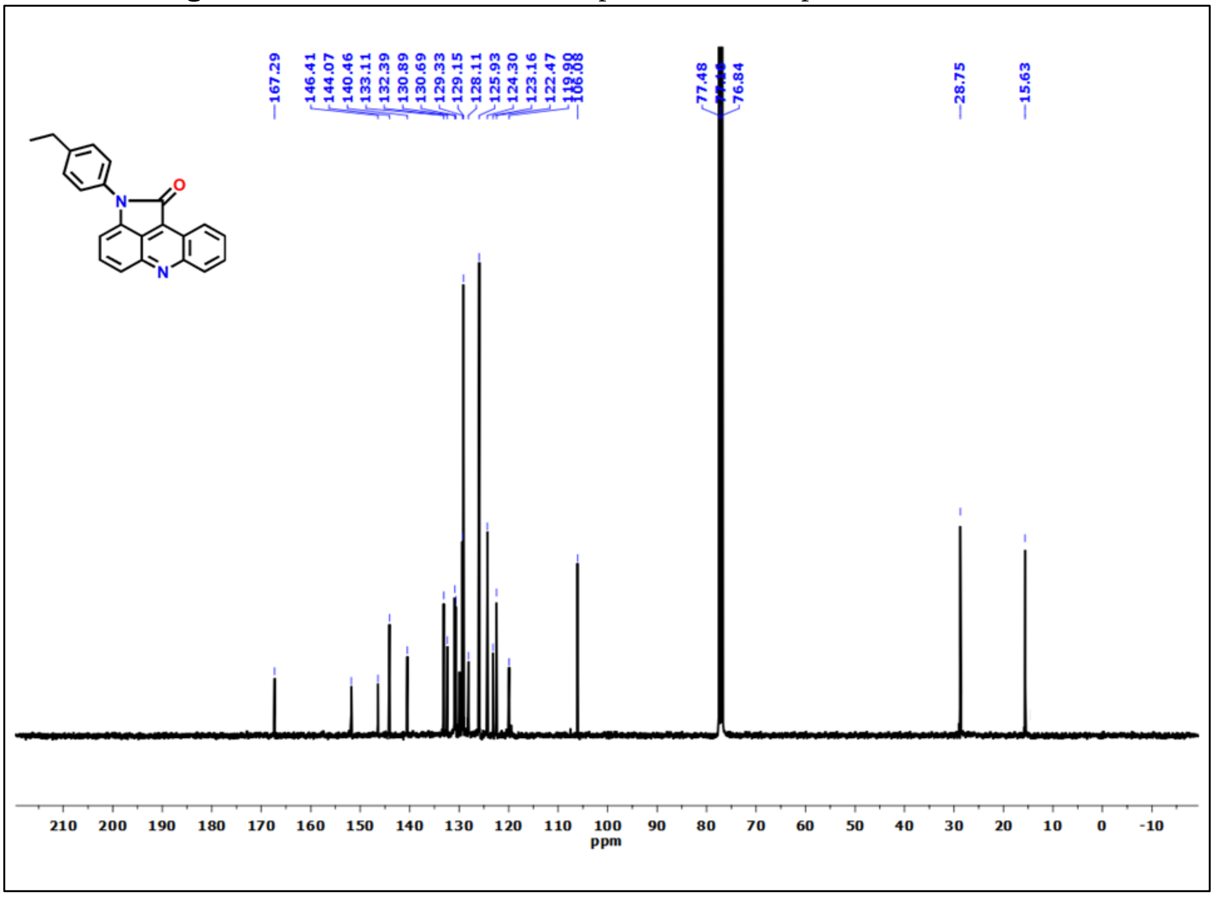

Figure S38: 100 MHz ^{13}C NMR spectrum of compound **7a** in $CDCl_3$

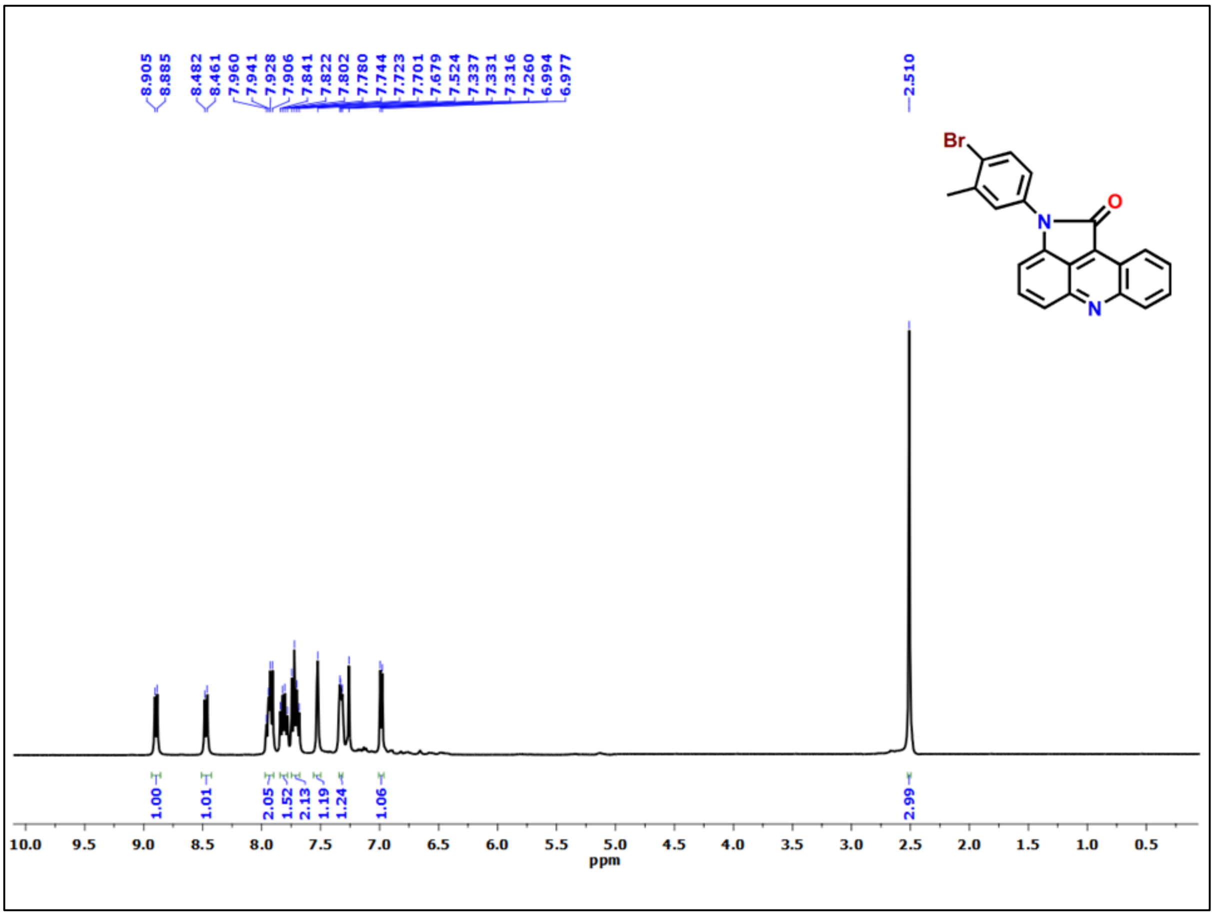

Figure S39: 400 MHz ^{1}H NMR spectrum of compound **7b** in $CDCl_3$

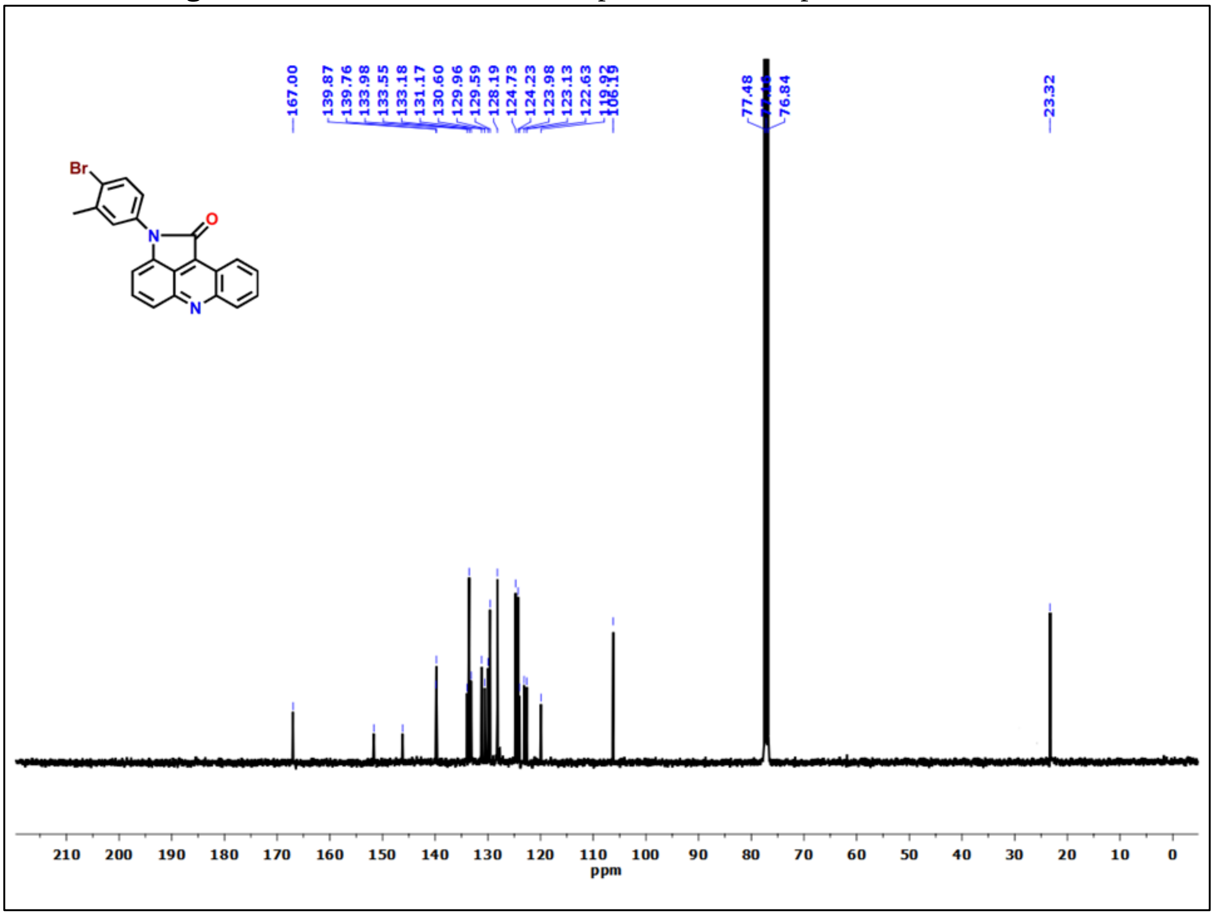

Figure S40: 100 MHz ^{13}C NMR spectrum of compound **7b** in $CDCl_3$

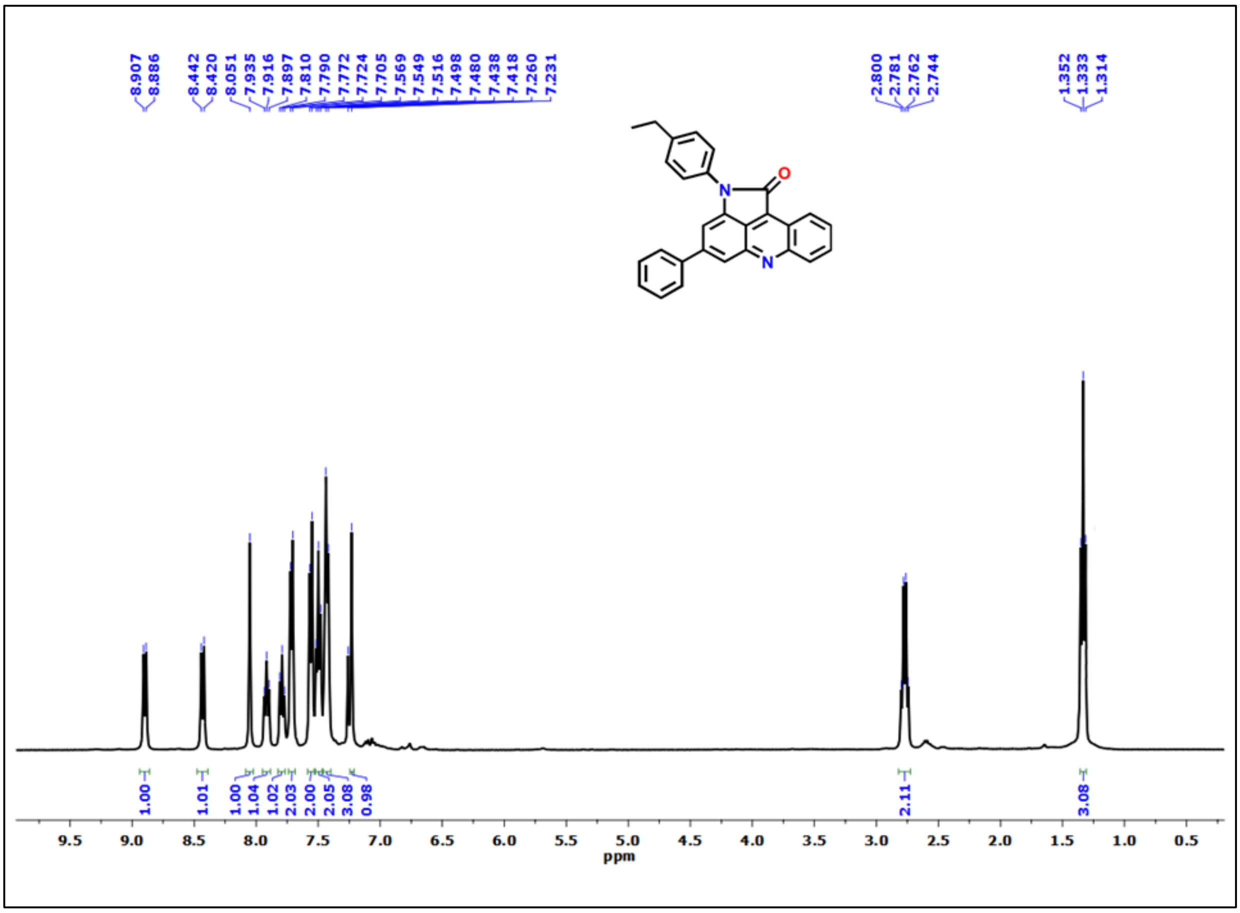

Figure S41: 400 MHz ^{1}H NMR spectrum of compound **7c** in $CDCl_3$

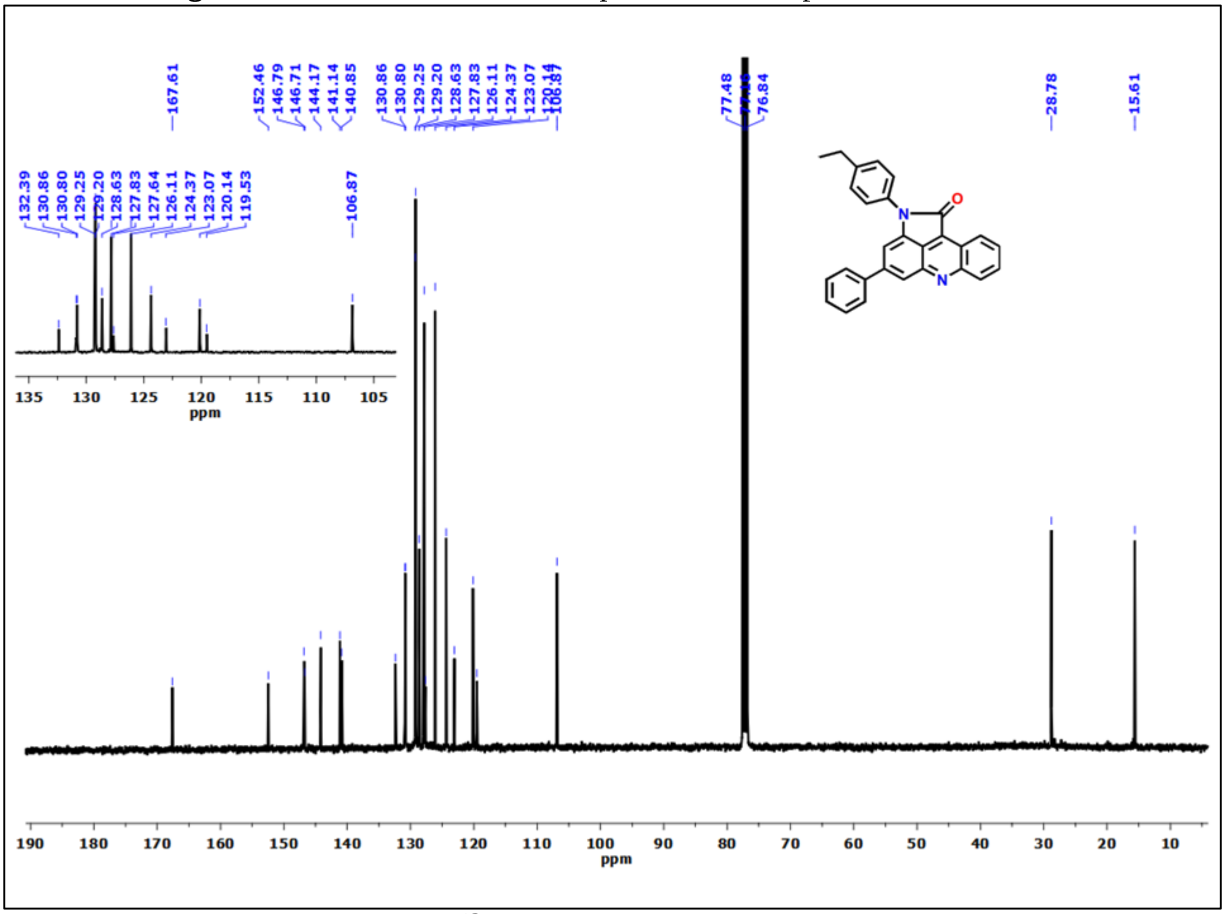

Figure S42: 100 MHz ^{13}C NMR spectrum of compound **7c** in $CDCl_3$

References

1. Sarkar, P.; Mukhopadhyay, C. *Green Chem.*, **2015**, *17*, 3452–3465. DOI: 10.1039/c5gc00156k.
2. Ray, S.; Bhaumik, A.; Pramanik, M.; Mukhopadhyay, C., *RSC Adv.*, **2014**, *4*, 15441–15450. DOI: 10.1039/c4ra01287a.
3. Wang, H.; Li, L.; Lin, W.; Xu, P.; Huang, Z.; Shi, D. *Org. Lett.*, **2012**, *14*, 4598-4601. DOI 10.1021/ol302058g.
